# Radiotherapy quality assurance program of ongoing clinical trial using stereotactic ablative radiation therapy for recurrent ovarian cancer (SABR-ROC): a dummy run study of a prospective, randomized, multicenter phase III trial (KGOG 3064/KROG 2204)

**DOI:** 10.1186/s12885-025-13892-9

**Published:** 2025-08-18

**Authors:** Sangjoon Park, Hojin Kim, Chan Woo Wee, Young Seok Kim, Jin Hwa Choi, Yeon Sil Kim, Jong Hoon Lee, Youngmin Choi, Jin Hee Kim, Yeona Cho, Hyun Ju Kim, Young Je Park, Won Park, Keun-Yong Eom, Yun Hwan Kim, Yong Bae Kim

**Affiliations:** 1 Department of Radiation Oncology, Yonsei University College of Medicine, 50 Yonsei-ro, Seodaemun-gu, Seoul, 03722 Republic of Korea; 2 Department of Radiation Oncology, Asan Medical Center, University of Ulsan College of Medicine, Seoul, Republic of Korea; 3 Department of Radiation Oncology, Chung-Ang University College of Medicine, Seoul, Republic of Korea; 4 Department of Radiation Oncology, College of Medicine, The Catholic University of Korea, Seoul, Republic of Korea; 5 Department of Radiation Oncology, St. Vincent’s Hospital, Catholic University of Korea, Suwon, Republic of Korea; 6 Department of Radiation Oncology, School of Medicine, Dong-A University, Busan, Republic of Korea; 7 Department of Radiation Oncology, Dongsan Medical Center, Keimyung University College of Medicine, Daegu, Republic of Korea; 8 Department of Radiation Oncology, Gangnam Severance Hospital, Yonsei University College of Medicine, Seoul, Republic of Korea; 9 Department of Radiation Oncology, Gachon University Gil Hospital, Incheon, Republic of Korea; 10 Department of Radiation Oncology, Korea University College of Medicine, Seoul, Republic of Korea; 11 Department of Radiation Oncology, Samsung Medical Center, Sungkyunkwan University School of Medicine, Seoul, Republic of Korea; 12 Department of Radiation Oncology, Seoul National University College of Medicine, Seoul National University Bundang Hospital, Seongnam, Republic of Korea; 13 Division of Gynecologic Oncology, Department of Obstetrics and Gynecology, Ewha Womans University College of Medicine, Seoul, Republic of Korea

**Keywords:** Recurrent ovarian Cancer, Stereotactic ablative radiation therapy, Radiation therapy planning, Dummy-Run, Quality assurance

## Abstract

**Background:**

Recurrent ovarian cancer is often treated with chemotherapy, but many patients experience multiple recurrences with progressively shorter intervals and poorer prognosis. Repeated chemotherapy reduces patients’ quality of life. Stereotactic Ablative Radiation Therapy for Recurrent Ovarian Cancer (SABR-ROC) (KGOG3064/KROG 2204) is an ongoing trial investigating the clinical efficacy of stereotactic ablative radiation therapy (SABR) for recurrent ovarian cancer. This study aimed to assess treatment planning consistency and protocol adherence in a prospective, randomized, multicenter phase III trial.

**Methods:**

In this dummy run study of a prospective, randomized, multicenter phase III trial (SABR-ROC), we examined the variability in target delineation, dose prescription, and treatment planning among 10 centers participating in the SABR-ROC trial. Four representative cases, each presenting with different anatomical sites and treatment challenges, were selected for evaluation. Target volume consistency was measured using the Dice similarity coefficient, and treatment plans were reviewed to follow predefined goals and constraints in the protocol.

**Results:**

Overall agreement in target delineation was low, with mean Dice similarity coefficients of 0.278 and 0.255 for gross tumor volume and planning target volume, respectively. Consistency was higher for cases involving lymph node and lung metastases but significantly lower for intraperitoneal and liver seeding metastases due to challenges in target delineation. Treatment plans generally adhered to protocol dose prescriptions, with minor deviations in planning target volume coverage, particularly in cases with multiple small metastases. Deviations from organ-at-risk constraints frequently occurred in cases involving small bowel proximity.

**Conclusions:**

This study highlights the challenges in standardizing SABR for recurrent ovarian cancer, particularly in achieving a consensus on target delineation and balancing treatment efficacy with organ-at-risk safety. Clinician discretion remains essential in complex cases. The insights from this study will guide the development of standardized protocols to improve outcomes and reduce adverse effects in patients with recurrent ovarian cancer.

**Trial registration:**

This trial was registered with ClinicalTrials.gov under the identifier NCT05444270 on June 29, 2022.

**Supplementary Information:**

The online version contains supplementary material available at 10.1186/s12885-025-13892-9.

## Background

Recurrent ovarian cancer is usually treated with chemotherapy; however, in many cases, the cancer recurs multiple times after treatment, with progressively shorter intervals between recurrences and the prognosis becoming poorer [1, 2]. Furthermore, repeated chemotherapy treatments lead to a reduced quality of life (QoL) in patients [3]. Recent studies have highlighted the radiosensitivity of epithelial ovarian cancer (EOC) cells, renewing interest in involved-field radiotherapy (IFRT) for recurrent tumors [4–9]. Based on these insights, the Korean Radiation Oncology Group (KROG) launched a prospective clinical trial in June 2022, titled “Stereotactic Ablative Radiation Therapy for Recurrent Ovarian Cancer (SABR-ROC).” The primary objective of this ongoing trial is to evaluate the efficacy of stereotactic ablative radiation (SABR) in combination with standard salvage therapy to improve disease-free survival (DFS) and assess post-treatment QoL in patients with recurrent EOC. In this trial, patients are randomized into two groups: a control group receiving standard salvage therapy and a treatment group receiving SABR in addition to standard salvage therapy [10]. The overall study scheme is presented in Supplementary Fig. 1 – Additional file 1.

Although the institutions participating in the SABR-ROC study possess advanced radiotherapy (RT) facilities and treatment planning systems, the allowance for SABR of various recurrent and metastatic sites in ovarian cancer can lead to different target definitions and RT delivery techniques. The variations in target volume determination and treatment planning among radiation oncologists emphasize the need for consistent treatment approaches across institutions. To address potential heterogeneity, it is crucial to evaluate and standardize the treatment planning processes [11]. To this end, a comprehensive radiotherapy quality assurance (RTQA) program has been developed for the SABR-ROC study.

As part of this effort, before patient enrollment began at participating centers, we aimed to evaluate and standardize treatment planning across institutions. Participating centers were provided with representative cases and independently delineated target volumes and prescribed doses to assess consistency in target volume delineation and dose prescription. Additionally, reference target volumes and dose prescriptions were provided for the standard cases, ensuring that institutions planned treatments based on predefined guidelines. This allowed for an independent evaluation of adherence to protocol guidelines, separate from the initial assessment of target volume delineation and dose prescription. Based on these findings, additional guidance and training programs were developed to further enhance standardization. This process aimed to minimize variability and ensure consistency in target volume delineation, dose prescription, and treatment planning for enrolled patients.

## Methods

### Case selection

In the SABR-ROC study, the treatment locations for recurrent ovarian cancer lesions are not restricted, allowing for the assessment of a variety of therapeutic scenarios. Four representative cases were selected based on recurrence patterns, tumor complexity, and clinical relevance for SABR. The cases included lymph node (LN) recurrence (retroperitoneal and supraclavicular LNs), lung metastases, and peritoneal seeding, reflecting common metastatic patterns in recurrent ovarian cancer. The selection also incorporated single vs. multiple lesions and varying anatomical challenges to evaluate target delineation feasibility and treatment planning variability. For each case, the reference target volume was established through a consensus panel at the headquarters center.

**Fig. 1:**
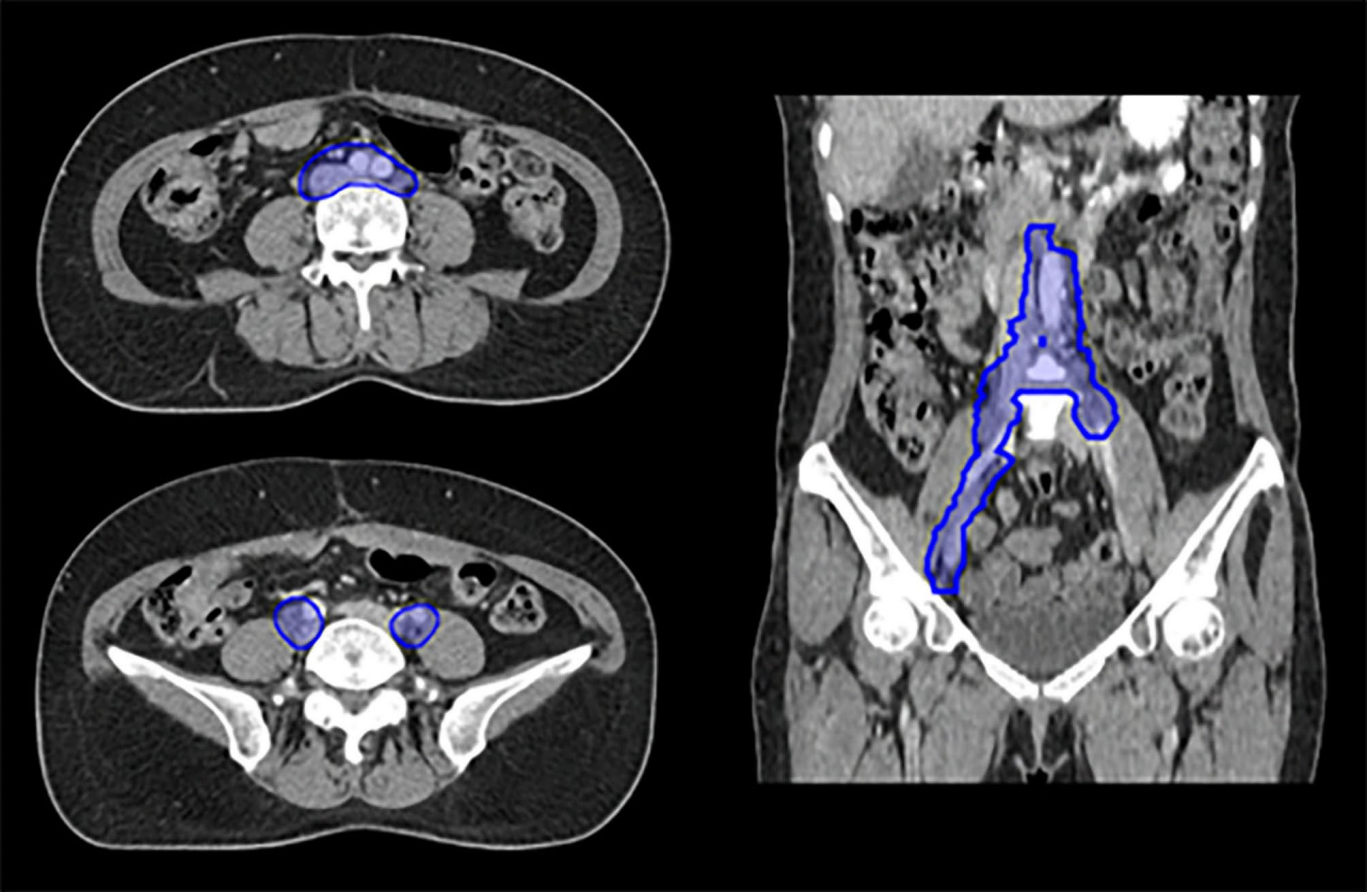
Target delineation for Case 1. Axial and coronal views of the planning target volume for a recurrent retroperitoneal lymph node

The patient in Case 1 was diagnosed with high-grade serous carcinoma of the ovary and initially treated with neoadjuvant chemotherapy (CTx), followed by debulking surgery and hyperthermic intraperitoneal chemotherapy (HIPEC). Recurrence was observed after multiple CTx. Positron emission tomography-computed tomography (PET-CT) after third-line treatment revealed partial remission with new lesions near the previously treated retroperitoneal LNs. The patient was referred for targeted salvage RT for the residual retroperitoneal LNs. Supplementary Fig. 2 – Additional file 1 presents the PET-CT scan at the time of salvage radiotherapy referral, showing residual retroperitoneal LN metastases following chemotherapy. Figure 1 illustrates the reference planning target volume for this case, as defined by the headquarters.

**Fig. 2:**
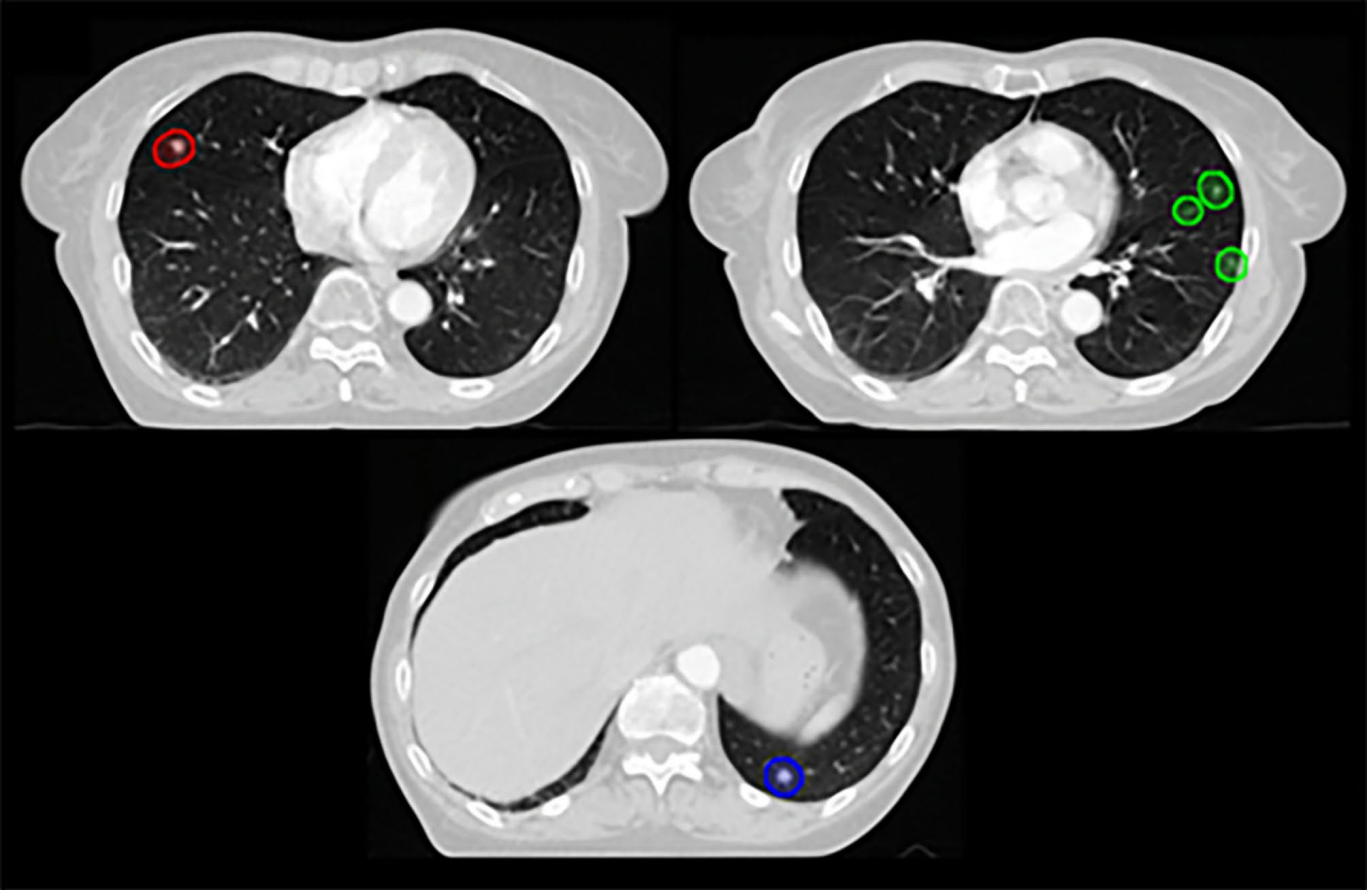
Target delineation for Case 2. Axial view of the planning target volumes for five small lung metastases

The patient in Case 2 was diagnosed with serous papillary ovarian adenocarcinoma and underwent initial debulking surgery. After multiple lines of systemic treatment addressing multiple recurrences in the mesentery, supraclavicular LN, mediastinal LN, and peritoneal cavity, multiple lung metastases and intraperitoneal seeding developed. The patient underwent a second debulking and right hemicolectomy, followed by adjuvant CTx, and was referred for subsequent RT for residual lung metastases. Supplementary Fig. 3 – Additional file 1 presents the chest CT scan at the time of radiotherapy referral, showing five metastatic lesions across both lungs. Figure 2 displays the reference target volume for this case.

**Fig. 3:**
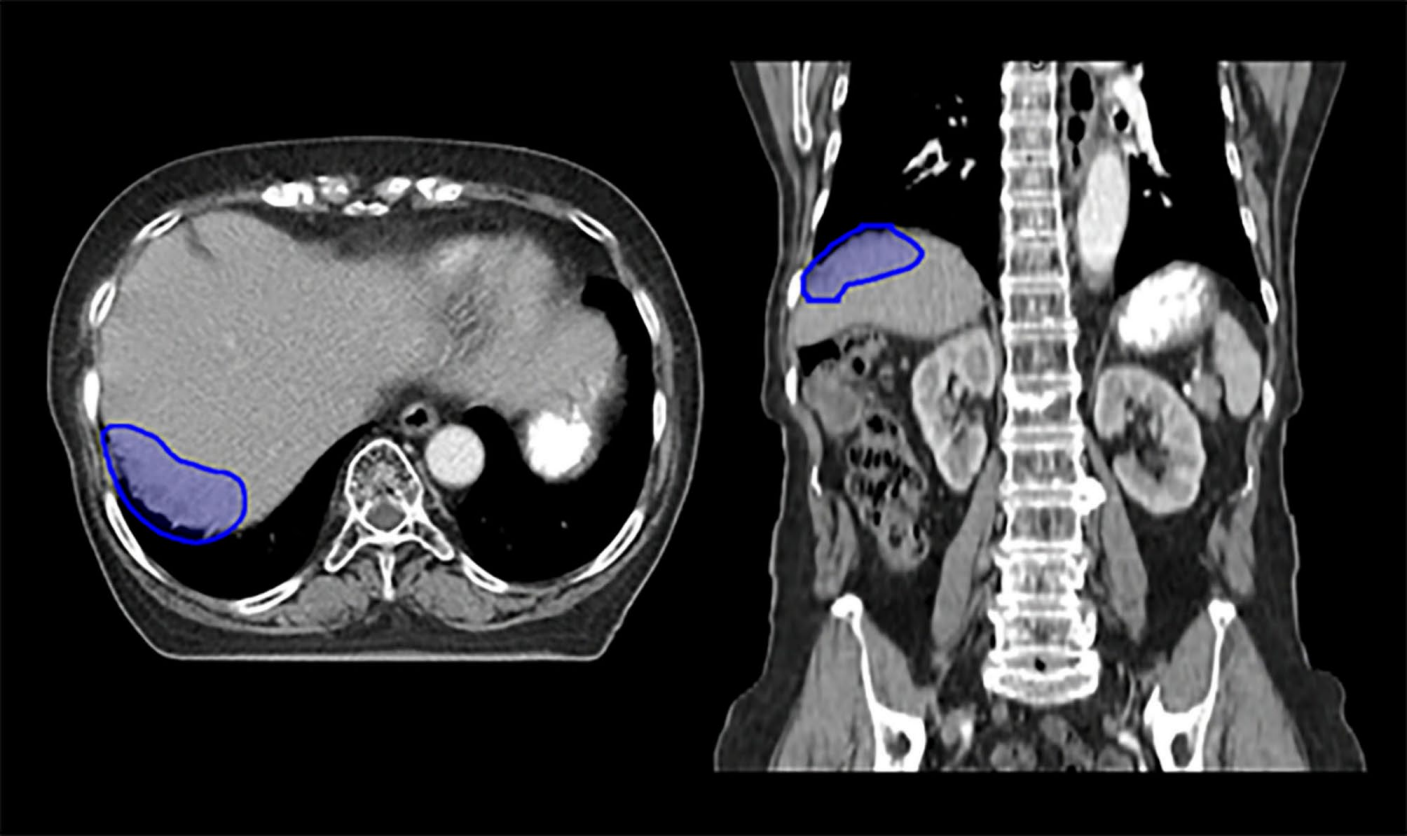
Axial and coronal views of the planning target volume for seeding metastases around the liver

The patient in Case 3 was initially treated with debulking surgery for papillary adenocarcinoma of the ovary and experienced multiple peritoneal metastases over time. After several lines of CTx, regrowth of seeding metastases around the liver was observed; the patient was therefore referred for salvage RT. Supplementary Fig. 4 – Additional file 1 presents the abdominopelvic CT scan at the time of radiotherapy referral, showing seeding metastases between the liver and diaphragm. Figure 3 illustrates the reference target volume for this case.

**Fig. 4:**
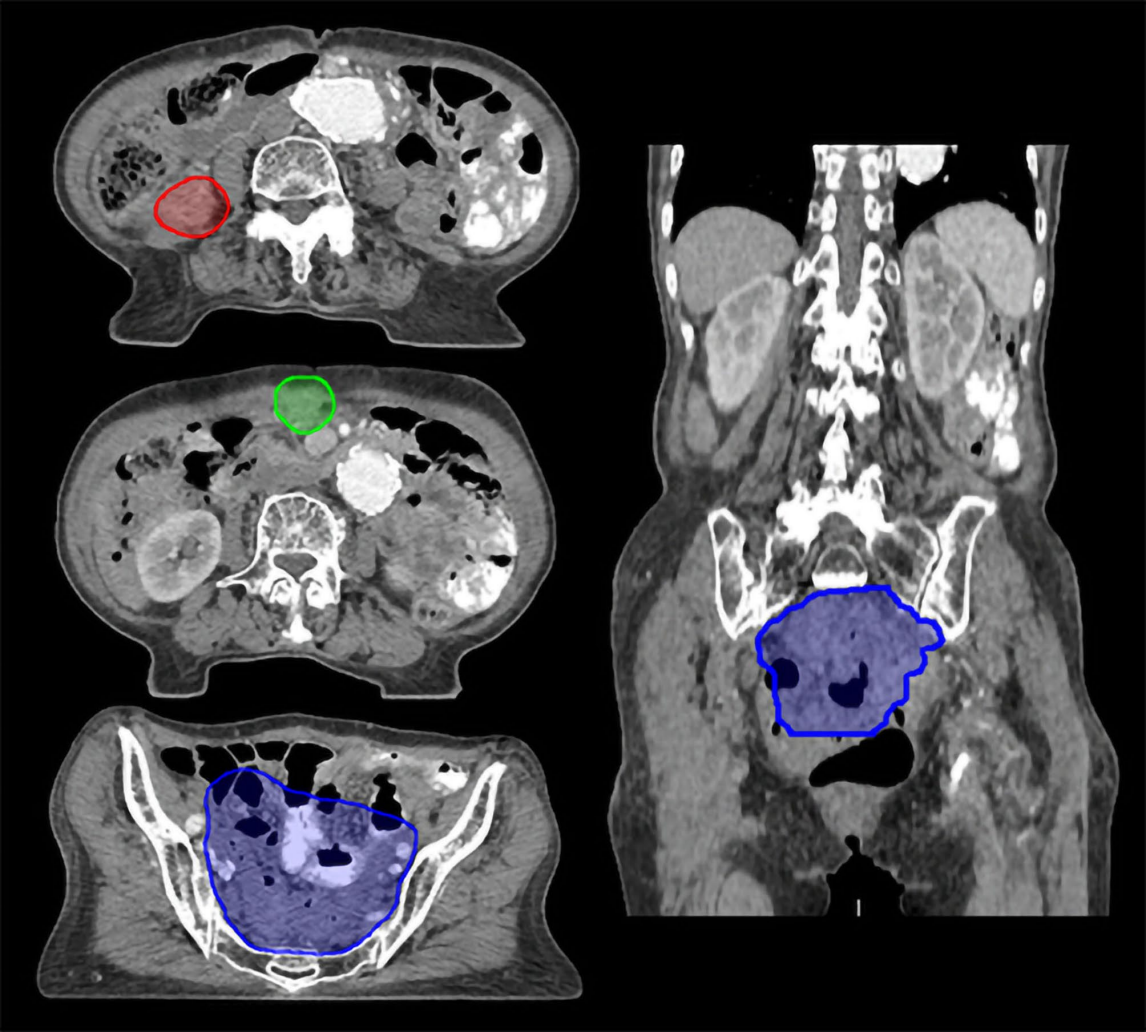
Axial and coronal views of the planning target volumes for intraperitoneal seeding metastases

The patient in Case 4 was diagnosed with high-grade serous carcinoma of the ovary with metastases to the supraclavicular LN and underwent neoadjuvant CTx followed by debulking surgery and adjuvant CTx. After multiple lines of systemic treatment for recurrence in the stomach, sigmoid colon, peritoneum, and subcarinal LN, there was an intraperitoneal recurrence, and the patient was referred for salvage RT for intraperitoneal lesions (Fig. 1D). Supplementary Fig. 5 – Additional file 1 presents the PET-CT scan at the time of radiotherapy referral, showing multiple intraperitoneal seeding metastase at the time of recurrence. Figure 4 shows the reference target volume for this case.

Further detailed information on these cases is provided in the *Details of Selected Cases* section of Supplementary Material (see Additional file 1).

### Process of the dummy run study

A total of 17 institutions were invited to participate in the dummy run study, which was conducted in two sequential phases to assess inter-institutional variability in target delineation, dose prescription, and treatment planning adherence.

In the first phase, four representative cases were selected, and de-identified Digital Imaging and Communications in Medicine (DICOM) format planning CT, PET-CT, and magnetic resonance imaging (MRI) scans related to diagnosis and major recurrence events were collected for each patient. These materials were shared via a network-attached storage (NAS) server accessible to all participating institutions. Each institution independently registered the provided planning CT images in their respective radiotherapy treatment planning (RTP) systems and delineated the gross tumor volume (GTV) and planning target volume (PTV) without predefined contours, following their own institutional practice patterns rather than strict adherence to the study protocol. The prescribed dose and dose scheme were also determined independently based on patient history and imaging studies. This phase aimed to assess real-world variability in target delineation and dose prescription across institutions, reflecting current clinical practice patterns of each institution. Each institution submitted DICOM RT structure files for all GTVs and PTVs, which were collected and analyzed to assess inter-institutional variability. Of the 17 invited centers, excluding the headquarters, 10 (58.8%) participated in this phase.

In the second phase, to mitigate the variations observed in the first phase and evaluate protocol adherence in treatment planning, standardized reference target volumes and dose prescriptions were provided by the headquarters. De-identified DICOM-format planning CT scans, along with headquarters-defined DICOM RT structure files for GTV and PTV, were exported from the MIM workstation (MIM Software Inc., Cleveland, OH, USA) and distributed to all participating centers via the NAS server. Each institution was required to generate treatment plans based on these predefined target volumes and dose prescriptions, ensuring compliance with the SABR-ROC study protocol. Unlike the first phase, this step allowed for an independent assessment of adherence to protocol-defined planning guidelines, separate from the variability in target delineation and dose prescription evaluated in the first phase. Researchers from each center submitted their final treatment plans, including DICOM RT structure files and radiation dose distributions, to the NAS. Additionally, a questionnaire on the prescribed dose scheme was completed and submitted (Additional file 2). The results from each institution were then analyzed to assess plan adherence to the standardized criteria. Of the 17 participating centers, 9 (52.9%) completed this phase.

Additional details of the participating centers for the targets and plans dummy run study are provided in Supplementary Tables 1 – Additional file 1.

### Evaluation

In the first phase, the consistency between the GTVs and PTVs across centers was assessed using the Dice similarity coefficient (DSC) to evaluate the inter-institutional variability in target delineation. As previously stated, some cases in the dummy run comprised multiple PTVs. We designated the sum of GTVs and the sum of PTVs delineated by the headquarters for each patient as the reference label. For each dummy run, the DSC was computed by comparing these reference labels to the sum of the GTVs and the sum of PTVs delineated by each participating institution. To further quantify inter-institutional agreement, pairwise DSCs were calculated across the 10 participating institutions for each of the four cases. Segmentation reliability was then assessed using Intraclass Correlation Coefficient (ICC) (2,1) from a two-way random-effects model with absolute agreement. To estimate the 95% confidence interval (CI) for DSC, we used the non-parametric bootstrap method with 1,000 resampling iterations with replacement, and the 95% CIs were derived from the relative frequency distribution.

The second stage involved reviewing the treatment plans generated by the participating institutions. Independent of the first stage of our dummy run, the same target volumes were provided to ensure that the participating institutions could accomplish the level of plan quality required for the clinical trial. During the review process, deviations from the PTV and organ-at-risk (OAR) dose constraints specified in the *Treatment Plan* section of the Supplementary Material (see Additional file 1) were identified and recorded, along with the indices in which the plan deviated. The deviations in the PTV were determined based on the PTV constraints. For the OARs, deviations were categorized as major or minor depending on whether the violation exceeded the tolerance by 1 Gy in dose and 3% in volume. Some dummy runs had multiple PTV volumes, which affected the steep fall-off gradient outside the PTV. In such cases, deviations in the ratio of 50% prescription isodose (R_50%_) and maximum dose at 2 cm (D_2cm_), were exempted to some extent (more detailed information is provided in Sect. 5 of Supplementary Material – Additional file 1).

## Results

### Target volume agreement

As shown in Table 1, the overall agreement levels were low, with a mean (standard deviation [SD]) DSC for all cases and centers of 0.278 (0.148) and 0.255 (0.143) for the GTV and PTV, respectively. Specifically, lower agreement was observed in Cases 3 and 4. For Case 3, the mean (SD) DSCs were 0.005 (0.013) and 0.108 (0.115) for GTV and PTV, respectively. For Case 4, these values were 0.199 (0.119) and 0.192 (0.178) for GTV and PTV, respectively. These cases had seeding lesions around the liver or intraperitoneal seeding metastases, where target delineation is challenging. In contrast, higher agreement levels were observed for Cases 1 and 2. For Case 1, the mean (SD) DSCs were 0.463 (0.187) and 0.315 (0.121) for GTV and PTV, respectively. For Case 2, these values were 0.515 (0.252) and 0.428 (0.197) for GTV and PTV, respectively. These cases had clearly visible lesions, such as lung lesions, lymph nodes, or nodal chains, where target delineation is more standardized. To further assess inter-institutional variability, ICC analysis was performed, revealing moderate consistency for GTV segmentation (ICC = 0.542) and low consistency for PTV segmentation (ICC = 0.299), indicating greater variability in PTV definitions across institutions.

**Table 1:** Dice similarity coefficient of each participating center calculated upon label

Case	Features	Participating center										
		A (95% CI)	C (95% CI)	D (95% CI)	E (95% CI)	F (95% CI)	G (95% CI)	H (95% CI)	J (95% CI)	K (95% CI)	L (95% CI)	Mean per case (STD)
Case 1	GTV	0.610 (0.514–0.707)	0.626 (0.529–0.723)	0.007 (0.000-0.100)	0.479 (0.382–0.578)	0.317 (0.217–0.418)	0.525 (0.424–0.614)	0.508 (0.401–0.617)	0.626 (0.536–0.722)	0.411 (0.311–0.504)	0.519 (0.422–0.617)	0.463 (0.187)
	PTV	0.384 (0.290–0.486)	0.431 (0.327–0.528)	0.049 (0.000-0.149)	0.288 (0.187–0.383)	0.235 (0.141–0.334)	0.304 (0.208–0.399)	0.366 (0.269–0.469)	0.334 (0.240–0.438)	0.491 (0.389–0.588)	0.270 (0.171–0.368)	0.315 (0.121)
Case 2	GTV	0.604 (0.503–0.708)	0.414 (0.312–0.523)	0.000 (0.000-0.095)	0.634 (0.539–0.734)	0.136 (0.042–0.230)	0.652 (0.555–0.748)	0.692 (0.590–0.787)	0.723 (0.631–0.819)	0.677 (0.579–0.770)	0.616 (0.519–0.711)	0.515 (0.252)
	PTV	0.516 (0.413–0.612)	0.401 (0.304–0.497)	0.001 (0.000-0.103)	0.559 (0.468–0.654)	0.174 (0.076–0.274)	0.507 (0.409–0.614)	0.545 (0.444–0.639)	0.618 (0.522–0.715)	0.566 (0.472–0.662)	0.393 (0.298–0.490)	0.428 (0.197)
Case 3	GTV	0.000 (0.000-0.092)	0.000 (0.000-0.101)	0.005 (0.000-0.105)	0.000 (0.000-0.095)	0.000 (0.000-0.097)	0.001 (0.000-0.093)	0.000 (0.000-0.098)	0.040 (0.000-0.141)	0.000 (0.000-0.099)	0.000 (0.000-0.097)	0.005 (0.013)
	PTV	0.106 (0.009–0.204)	0.227 (0.128–0.318)	0.045 (0.000-0.144)	0.000 (0.000-0.100)	0.002 (0.000-0.096)	0.180 (0.081–0.279)	0.000 (0.000-0.095)	0.301 (0.198-0.400)	0.000 (0.000-0.098)	0.215 (0.121–0.319)	0.108 (0.114)
Case 4	GTV	0.307 (0.205–0.404)	0.228 (0.126–0.332)	0.000 (0.000-0.095)	0.073 (0.000-0.174)	0.090 (0.000-0.189)	0.192 (0.099–0.283)	0.324 (0.228–0.420)	0.373 (0.280–0.475)	0.237 (0.131–0.332)	0.168 (0.076–0.269)	0.199 (0.119)
	PTV	0.187 (0.097–0.288)	0.504 (0.404-0.600)	0.031 (0.000-0.125)	0.019 (0.000-0.124)	0.021 (0.000-0.111)	0.329 (0.230–0.429)	0.247 (0.142–0.346)	0.039 (0.000-0.137)	0.122 (0.026–0.221)	0.418 (0.314–0.518)	0.192 (0.178)
**Mean per center** **(STD**)	GTV	0.380 (0.290)	0.317 (0.267)	0.003 (0.004)	0.297 (0.308)	0.136 (0.133)	0.343 (0.299)	0.381 (0.295)	0.441 (0.305)	0.331 (0.285)	0.326 (0.290)	0.278 (0.148)
	PTV	0.298 (0.186)	0.391 (0.117)	0.032 (0.022)	0.217 (0.264)	0.108 (0.114)	0.330 (0.135)	0.290 (0.229)	0.323 (0.237)	0.295 (0.276)	0.324 (0.097)	0.255 (0.143)

Abbreviations: STD, standard deviation; GTV, gross tumor volume; PTV, planning target volume

Values are presented as Dice similarity coefficients

This observed variability in target volume definitions among different organs reflects differences in institutional practices prior to standardization and prompted us to distribute a standardized reference target volume for the analysis of dose prescriptions and planning QA in the second stage.

### Dose prescription agreement

Table 2 provides the results of the dose prescriptions across the participating centers. While dose prescriptions generally followed protocol guidelines, variations were observed across institutions. In scenarios involving the treatment of targets near radiosensitive OAR, as in Cases 1 and 4, most centers opted for the protocol with the largest fraction number (10 fractions). Conversely, when treating lesions, such as metastatic lung nodules or seedings around the liver, where higher doses can be safely delivered, a regimen of 3–5 fractions was commonly employed.

**Table 2:** Comparison of dose prescriptions by participating centers for each case

Case	Features	volume	Participating center																							
			A		B		C		E		F		H		I		K		L		M (headquarter)		Mean (STD)			
			TD	fx	TD	fx	TD	fx	TD	fx	TD	fx	TD	fx	TD	fx	TD	fx	TD	fx	TD	fx	TD	fx		
Case 1	PTV1	165 cc	45	10	35	10	40	10	40	10	40	10	35	10	40	10	30	5	40	10	35	10	38.000 (4.216)	9.500 (1.581)		
Case 2	PTV1	3 cc	40	5	30	3	33	3	35	5	30	3	35	5	30^*^	3^*^	30^*^	3^*^	30	3	40	5	33.300 (4.084)	3.800 (1.033)		
	PTV2	10.5 cc	40	5	35	5	33	3	35	5	30	3	35	5					30	3	40	5	34.750 (3.845)	4.250 (1.035)		
	PTV3	7.5 cc	40	5	30	3	33	3	35	5	30	3	35	5					30	3	40	5	34.125 (4.190)	4.000 (1.069)		
Case 3	PTV1	49 cc	40	5	35	5	35	5	35	5	35	5	27	3	35	5	35	5	30	3	24	3	33.100 (4.701)	4.400 (0.966)		
Case 4	PTV1	16 cc	35	10	40	10	40	10	40	10	40	10	35	10	40^*^	10^*^	30^*^	5^*^	40	10	24	3	36.400 (5.542)	8.800 (2.573)		
	PTV2	16 cc	35	10	40	10	40	10	35	10	40	10	35	10					40	10	24	3	36.125 (5.489)	9.125 (2.475)		
	PTV3	544 cc	35	10	40	10	40	10	35	10	40	10	35	10	35	10	25	5	37	10	35	10	35.700 (4.398)	9.500 (1.581)		

Abbreviations: TD, total dose; fx, fraction; PTV, planning target volume

Values are presented in Gy / fractions

^*^ Dose prescribed in a single plan for multiple target volumes

The greatest variability in dose prescription occurred in Case 4, involving intraperitoneal seeding metastases, likely due to differences in clinical judgment. As physicians had to balance tumor coverage with minimizing radiation exposure to adjacent radiosensitive organs, particularly the small bowel, the number of fractions varied more than in other cases.

### Treatment plan review

The results of compliance with the PTV dosimetry regulations are presented in Table 3. Overall, the most frequent cause of noncompliance was inadequate PTV coverage, followed by deviations in R_50%_ and D_2cm_, respectively. The results of compliance with the OAR constraint regulations are presented in Table 4. Noncompliance occurred most frequently with constraints related to the small bowel, and an overdose to the duodenum was also observed in some centers.

**Table 3:** Comparison of compliance with PTV dosimetry regulations for each case

Case	Features		Participating center									
			**A**	**B**	**C**	**E**	**F**	**H**	**I** ^*^	**K** ^*^	**L** ^†^	**M** ^†^ **(headquarter)**
Case 1	PTV1^‡^	Deviations	0	1	0	0	0	0	0	0	1	1
		Parameters	-	PTV coverage	-	-	-	-	-	-	PTV coverage	PTV coverage
Case 2	PTV1	Deviations	0	3	2	3	0	2	2	1	2	0
		Parameters	-	PTV coverage R_50%_ D_2cm_	R_50%_ (major) D_2cm_ (major)	High-dose Spillage R_50%_ D_2cm_	-	R_50%_ D_2cm_	R_50%_ D_2cm_	R_50%_	PTV coverage R_50%_	-
	PTV2^‡^	Deviations	0	1	0	0	0	0	0	0	0	0
		Parameters	-	PTV coverage	-	-	-	-	-	-	-	-
	PTV3	Deviations	0	2	1	1	0	1	0	0	0	0
		Parameters	-	PTV coverage R_50%_	R_50%_ (major)	R_50%_	-	R_50%_	-	-	-	-
Case 3	PTV1	Deviations	0	2	0	1	0	0	0	1	0	0
		Parameters	-	PTV coverage R_50%_	-	R_50%_	-	-	-	D_2cm_ (major)	-	-
Case 4	PTV1	Deviations	1	3	0	0	0	0	1	0	0	0
		Parameters	PTV coverage	PTV coverage R_50%_ D_2cm_	-	-	-	-	D_2cm_	-	-	-
	PTV2	Deviations	0	2	0	0	0	0	0	0	0	0
		Parameters	-	PTV coverage R_50%_	-	-	-	-	-	-	-	-
	PTV3	Deviations	2	2	0	2	1	2	2	2	0	1
		Parameters	PTV coverage R_50%_	PTV coverage R_50%_	-	PTV coverage R_50%_	PTV coverage (major)	PTV coverage R_50%_	R_50%_ D_2cm_ (major)	PTV coverage, R_50%_	-	R_50%_

Abbreviations: PTV, planning target volume

^*^ Centers adopting PTV-EVAL in planning for all PTVs in Case 4

^†^ Centers adopting PTV-EVAL in planning of PTV3 in Case 4

^‡^ PTV contains multiple target volumes; thus, the R_50%_ and D_2cm_ deviations are exempted

**Table 4:** Comparison of compliance with normal organ dosimetry regulation for each patient

Case	Features		Participating center									
			**A**	**B**	**C**	**E**	**F**	**H**	**I** ^*^	**K** ^*^	**L** ^†^	**M** ^†^ **(headquarter)**
Case 1	PTV1	Deviations	1	0	1	1	0	0	0	2	1	0
		OARs	Small bowel Dmax	-	Small bowel Dmax (minor)	Small bowel Dmax (minor)	-	-	-	Duodenum Dmax (minor) Duodenum 10 cc (minor)	Small bowel Dmax (major)	-
Case 2	PTV1	Deviations	0	0	0	0	0	0	0	0	0	0
		OARs	-	-	-	-	-	-			-	-
	PTV2	Deviations	0	0	0	0	0	0			0	0
		OARs	-	-	-	-	-	-	-	-	-	-
	PTV3	Deviations	0	0	0	0	0	0			0	0
		OARs	-	-	-	-	-	-			-	-
Case 3	PTV1	Deviations	0	0	0	0	0	0	0	0	0	0
		OARs	-	-	-	-	-	-	-	-	-	-
Case 4	PTV1	Deviations	0	0	0	0	0	0	0	0	0	0
		OARs	-	-	-	-	-	-			-	-
	PTV2	Deviations	0	0	0	0	0	0		-	1	0
		OARs	-	-	-	-	-	-	-		Small bowel Dmax (major)	-
	PTV3	Deviations	0	1	2	0	2	0		0	2	0
		OARs	-	Small bowel 120 cc (major)	Small bowel Dmax (major) Small bowel 120 cc (major)	-	Small bowel Dmax (minor) Small bowel 120 cc (major)	-		-	Small bowel Dmax (major) Small bowel 120 cc (major)	-

Abbreviations: PTV, planning target volume; OAR, organ at risk

^*^ Centers adopting PTV-EVAL in planning for all PTVs in Case 4

^†^ Centers adopting PTV-EVAL in planning of PTV3 in Case 4

In the patient in Case 1, who required treatment of retroperitoneal LNs, compliance with PTV dosimetry was largely observed, except for some deviations in the PTV coverage. However, when exemptions for multiple volume deviations in R_50%_ and D_2cm_ were not granted, R_50%_ deviations were reported by nearly all centers, indicating challenges in meeting moderate-dose spillage parameters when treating multiple target volumes with a single plan. Supplementary Tables 2 – Additional file 1 presents the outcomes without these exemptions. In terms of the OAR constraints, the dose constraint for the small bowel was most frequently exceeded.

In the patient in Case 2, who required treatment of five multiple but small lung nodules, there were no deviations in the OAR constraints. However, deviations in R_50%_ and D_2cm_ were the most frequently observed in all cases. In particular, in Case 2, when PTV was evaluated without considering multiple target volumes, deviations in R_50%_ and D_2cm_ were observed in most centers, demonstrating the challenges in treating multiple volumes. The results without consideration of multiple target volumes are provided in more detail in Supplementary Tables 2 – Additional file 1.

In the patient in Case 3, who required treatment of seeding around the liver, treatment planning was relatively straightforward because the target was primarily surrounded by parallel OARs, such as the lungs and liver, with no other critical organs in proximity. Consequently, apart from a single instance of deviation in the PTV coverage, compliance with dosimetry regulations was generally excellent in terms of both the PTV and OAR constraints.

In the patient in Case 4, with multiple intraperitoneal seeding metastases, the highest incidence of noncompliance was reported. Participating centers often face a challenge in achieving adequate PTV coverage that exceeds the constraints for OARs. Conversely, strict adherence to OAR constraints frequently results in significantly compromised PTV coverage. Therefore, as shown in Supplementary Fig. 6 – Additional file 1, we introduced PTV-EVAL by subtracting OARs to enhance planning feasibility for cases like Case 4, which was adopted by Centers 7, 8, 9, and 10. Among the six centers that planned using conventional PTV, inadequate PTV coverage was observed in five centers (83.3%), and small bowel constraints were exceeded in three centers (50%). However, among the four centers that used PTV-EVAL, only one (25.0%) reported inadequate PTV coverage and only one (25.0%) exceeded the small bowel constraints.

## Discussion

This dummy run study of the SABR-ROC prospective study assessed treatment planning consistency and protocol adherence and revealed significant variability in target volume delineation and treatment planning among participating centers.

IFRT has historically been underutilized in the management of ovarian cancer but has recently been revived as a salvage therapy for recurrent disease. Despite its efficacy in tumor control [12, 13], IFRT with a conventional dose scheme necessitates a prolonged treatment duration of approximately 5 weeks, during which it is challenging to administer the cytotoxic CTx. To mitigate these challenges, the SABR-ROC study advocates the adoption of SABR as an efficacious alternative without compromising treatment efficacy. Even at the maximum permitted fraction count, SABR can be completed within two weeks, which is significantly shorter than conventional IFRT and thereby reduces interruptions to systemic therapy. Moreover, SABR substantially reduces the overall period of RT and minimizes radiation exposure to surrounding healthy tissues with a better dose fall-off profile, facilitating subsequent re-irradiation, if needed [14–18].

Despite its potential advantages, RT, particularly SABR, is yet to become a standard treatment for ovarian cancer. Many gynecologic oncologists do not refer patients for RT, leading to a limited experience among radiation oncologists specializing in gynecological cancers. Consequently, there is a lack of consensus regarding the target delineation and treatment planning for recurrent ovarian cancer. Salvage RT for metastatic ovarian cancer presents unique challenges, particularly for peritoneal metastases, which are the most common sites of metastasis [19]. Radiosensitive OARs, such as the small bowel, are often in close proximity, complicating treatment planning and resulting in significant variability in target volume delineation. This necessitates careful clinical discretion.

Although the prospective SABR-ROC study provides detailed protocols, considerable heterogeneity was observed in target volume delineation, dose prescription, and planning across centers. Notably, significant variations were observed in the delineation of poorly visible and ill-defined seeding lesions compared to more standardized targets, such as nodal chains or clearly discernible lung nodules. A pre-study workshop was conducted to educate the participants on protocol-defined target delineation; however, the results were unsatisfactory. The lack of consensus guidelines and the selection of cases with less clear definitions likely contributed to the differences in interpretation among radiation oncologists. Additionally, providing only a single time-point image limits the ability to define tumors based on sequential imaging changes. Furthermore, while the protocol referenced existing studies on target volume definitions, participants with limited SABR experience may have found strictly adhering to these guidelines challenging.

In planning, the most frequently deviated parameters were conventional moderate-dose spillage parameters in SABR, such as R_50%_ and D_2cm_. These deviations were primarily observed in cases with multiple adjacent lesions, where contouring uncertainty and proximity to radiosensitive organs posed challenges. While SABR is technically feasible for most recurrent ovarian cancer cases, certain lesions with complex anatomical features may require modified treatment approaches rather than adjustments to existing SABR constraints. Case-specific analysis revealed that lesions involving radiosensitive OARs, such as those in Case 4, often necessitated trade-offs between adhering to OAR constraints and achieving optimal PTV dosimetry. This emphasizes the importance of clinician discretion in treatment planning, particularly in complex scenarios, such as intraperitoneal seeding, where treatment approaches may vary significantly among clinicians. The outcomes of this study emphasize the need for consensus on prescription and constraint standards based on patient outcomes to facilitate the effective use of IFRT.

## Conclusions

This study aimed to enhance the precision and effectiveness of treatments for patients with recurrent ovarian cancer by leveraging the latest SABR technique. The findings from this dummy run of the SABR-ROC prospective study emphasize the complexities inherent in standardizing target volume delineation and treatment planning in SABR for metastatic ovarian cancer. These challenges highlight the indispensable role of clinician judgment in navigating the intricacies of individual patient cases. We hope that the results of this study will offer insights that will aid in the development of standardized protocols for the application and planning of SABR for ovarian cancer, potentially improving treatment outcomes and reducing adverse effects.

## Electronic supplementary material

Below is the link to the electronic supplementary material.

Supplementary Material 1

Supplementary Material 2

## Data Availability

The data that support the findings of this study are available from the principal investigators of each institution that provided the dummy run radiation therapy plans, but restrictions apply to the availability of these data, which were used under license for the current study, and so are not publicly available. However, data are available from the authors upon reasonable request and with permission from the principal investigators after the formal consent.

## Abbreviations

SABR: Stereotactic Ablative Radiation Therapy
ROC: Recurrent Ovarian Cancer
EOC: Epithelial ovarian cancer
IFRT: Involved-field radiotherapy
DFS: Disease-free survival
RT: Radiotherapy
RTQA: Radiotherapy quality assurance
CTx: Chemotherapy
HIPEC: Hyperthermic intraperitoneal chemotherapy
PET-CT: Positron emission tomography-computed tomography
LN: Lymph node
DICOM: Digital Imaging and Communications in Medicine
MRI: Magnetic resonance imaging
NAS: Network-attached storage
RTP: Radiotherapy treatment planning
GTV: Gross tumor volume
PTV: Planning target volume
DSC: Dice similarity coefficient
OAR: Organ-at-risk
SD: Standard deviation
