# Supplementary material for: Radiotherapy quality assurance program of ongoing clinical trial using stereotactic ablative radiation therapy for recurrent ovarian cancer (SABR-ROC): a dummy run study of a prospective, randomized, multicenter phase III trial (KGOG 3064/KROG 2204)
Source: BMC Cancer. 2025 Aug 18;25:1336. doi: 10.1186/s12885-025-13892-9 (PMC12362936; doi:10.1186/s12885-025-13892-9)
Supplement: Supplementary file 1 — Supplementary Material 1 [file 12885_2025_13892_MOESM1_ESM.pdf]

[SABR-ROC Planning Dummy-run Questionnaire]

Please fill in the prescribed dose and related information for each case.

If there are any specific notes related to the plan, please enter them in the [Remarks] section below each case.

Institution:

1. **Case #1 (F/51)**
   A. Number of field: 1 (PALN ~ iliac LN)
   B. GTV1: PALN ~ iliac LN
   C. PTV1: GTV1 + margin

Target	Dose per fraction (cGy)	Total fraction	Total dose (cGy)
PTV1			

[Remarks]

2. **Case #2 (F/69)**
   A. Number of field: 3 (RLL / LUL / LLL)
   B. GTV1: RLL nodule (1개)
   C. PTV1: GTV1 + margin
   D. GTV2: LUL nodule (3개)
   E. PTV2: GTV2 + margin
   F. GTV3: LLL nodule (1개)
   G. PTV3: GTV3 + margin

Target	Dose per fraction (cGy)	Total fraction	Total dose (cGy)
PTV1			=
PTV2			
PTV3			

[Remarks]

4. Case #3 (F/73)

A. Number of field: 1 (subphrenic~perihepatic lesion)

B. GTV1: subphrenic~perihepatic

C. PTV1: GTV1 + margin

**Target**	**Dose per fraction (cGy)**	**Total fraction**	**Total dose (cGy)**
PTV1			=

[Remarks]

5. Case #4 (F/71)

A. Number of field: 3 (Paracolic / abdominal wall / pelvic seeding)

B. GTV1: paracolic seeding

C. ITV1: GTV1 + internal margin

D. PTV1: GTV1 + margin

E. GTV2: abdominal wall mass

F. ITV2: GTV2 + internal margin

G. PTV2: GTV2 + margin

H. PTV3: pelvic seeding (contoured as PTV due to absence of a clear GTV)

**Target**	**Dose per fraction (cGy)**	**Total fraction**	**Total dose (cGy)**
PTV1			=
PTV2			
PTV3			

[Remarks]
