# Supplementary material for: Radiotherapy quality assurance program of ongoing clinical trial using stereotactic ablative radiation therapy for recurrent ovarian cancer (SABR-ROC): a dummy run study of a prospective, randomized, multicenter phase III trial (KGOG 3064/KROG 2204)
Source: BMC Cancer. 2025 Aug 18;25:1336. doi: 10.1186/s12885-025-13892-9 (PMC12362936; doi:10.1186/s12885-025-13892-9)

**Supplementary Materials**

**1. Radiation Therapy**

**2. Technical Aspects**

**3. Localization, Simulation, and Fixation**

**4. Target Volume**

**5. Treatment Plan**

**6. Critical Structures**

**7. Dose-Volume Limit to Normal Organs**

**8. Compliance Criteria**

**9. Details of Selected Cases**

**Supplementary Table 1. Submission Status of Targets and Plans During Dummy Runs Across Participating Centers**

**Supplementary Table 2. Comparison of Compliance with PTV Dosimetry Regulations for Each Case Without Considering Multiple Target Volume for Single Plan**

**Supplementary Figure 1. Study Scheme of the SABR-ROC**

**Supplementary Figure 2. PET-CT scan at the time of salvage radiotherapy referral for Case 1, showing residual retroperitoneal LN metastases following chemotherapy.**

**Supplementary Figure 3. Chest CT scan of Case 2. Showing five metastatic lesions across both lungs.**

**Supplementary Figure 4. Abdominopelvic CT scan of Case 3, showing seeding metastasis regrowing between the liver and diaphragm.**

**Supplementary Figure 5. PET-CT scan of Case 4, showing multiple intraperitoneal seeding at the time of the salvage radiotherapy referral**

**Supplementary Figure 6. Comparison between PTV and PTV-EVAL. (A) The Planning Target Volume (PTV) is highlighted in blue. (B) PTV-EVAL, after subtracting organ-at-risk.**

**1. Radiation Therapy**

SABR on the metastatic site should be completed within two weeks of the first day of SABR initiation for three and five treatments; within three weeks for ten treatments. In the case of three and five treatments, the treatment is recommended to be administered every other day, while daily treatment is recommended for ten treatments. Not all treatment sites are required to be irradiated on the same day.

The goal of SABR is to target metastatic tumors precisely and minimize exposure to surrounding normal organs. As there are various methods to perform SABR, any methods with a required credential can be applied. Most commercially available X-ray photon therapy machines are all acceptable. For example, all common linear accelerators or special linear accelerators with an image guidance device (e.g., Novalis, Trilogy, Versa, Artiste, TrueBeam) are permitted. Using such therapy machines, radiation therapy based on intensity control is performed. Special therapy devices, including CyberKnife or Tomotherapy, can also be used if they have passed credentials and met all technical requirements prescribed in the protocol. When using a conventional linear accelerator without image-guided radiation therapy (IGRT), the accelerator should be equipped with a CT scanner in the treatment which can act as an image guidance device. Conformal radiotherapy is not permitted.

IGRT is required for this study. IMRT, including VMAT, may have uncertainties due to its characteristics of dosimetry if there is tumor movement that is not properly considered. If the tumor or the treatment target is near the thorax, the movement of the tumor should be controlled appropriately, and a treatment plan specific to each target site should be established. Breathing control techniques (e.g., Active Breathing Control, Deep Inspiration Breath Holding technique, etc.) to control the tumor movement can be used.

Dose Fractionation

| **Number of Fractions** | **Preferred Doses** | **Acceptable Doses** |
| --- | --- | --- |
| 1 | 20 Gy | 16-24 Gy |
| 3 | 30 Gy | 24-33 Gy |
| 5 | 35 Gy | 25-40 Gy |
| 10 | 40 Gy | 35-45 Gy |

In the case of 6-9 fractions, the prescribed doses of 5 fractions and 10 fractions along with dose constraint are applied proportionally.

Within acceptable doses, each institution may use its standard dose according to the clinical situation.

The prescribed dose should be at least 95% of the PTV. It can be lowered to 70% only if the PTV includes a normal organ (serial structure) or is immediately adjacent to the organ. However, all efforts should be made to cover 100% of the GTV with the prescribed dose at the lower limit of the acceptable dose.

NOTE: Metastatic lesions in completely different anatomical locations of the same patient can be treated with a combination of different therapeutic doses. For example, it is possible to treat a central lung metastasis with 35 Gy in 5 factions, while treating a spinal metastasis with 20 Gy in 1 fraction. Nevertheless, the same dose combination for all metastases is preferred, which may facilitate the dose-volume analysis for normal tissue. Treatments for each metastatic site should be performed at a 24-hour interval at least. However, if the treatment is given to different sites, it can be performed on consecutive days.

**2. Technical Aspects**

Physical aspects

In terms of energy, only an X-ray photon beam of 6 MV or more is permitted. As for tumors within 3 cm of the lung, a photon beam of 6-10 MV is required. Radiation therapies using particles other than photons such as Cobalt- 60, electron, proton, or heavy ion are not permitted.

In the case of a central or peripheral metastasis in the lung, a photon beam of 10 MV or more may be applied when the cumulative distance of the soft tissue is longer than or equal to 10 cm, or when the tumor is attached to the thoracic or abdominal wall and the skin dose needs to be reduced.

Treatment technique

This study requires photon beam therapy: both IMRT and VMAT are acceptable, and tomotherapy and cyberknife can be used.

**3. Localization, Simulation, and Fixation**

Fixation

The patient should be positioned in a location where the patient feel comfortable and there is no uncontrolled movement during treatment. It is not recommended to place the patient on the couch directly and rely only on image guidance.

Simulation

A CT-based treatment plan should be established in all patients. When fiducial markers are used, images should be taken under a setting that include all ranges. High-resolution CT scan images should be used, and CT slices should be 3 mm in thickness or smaller. Multiple CT scans are also permitted if there are two different treatment sites in which images cannot be taken at once. It is recommended that all treatment sites be treated under a single position. However, the patient’s position can be altered in cases when radiotherapy is delivered to where the lungs and extremities.

Use of a contrast

In the case of liver metastases, an IV contrast is required. For other metastatic sites, the IV contrast is also recommended but may be replaced with other contrasts at the discretion of the attending physician.

Evaluation and control of breathing movement

All metastases that can be move by the patient’s breathing should be evaluated with 4D CT, fiducial marker, and fluoroscopy. In addition, for metastatic cancer with movement of 5 mm or greater by respiration, breathing control techniques, including an abdominal compression device, active breathing control, breathing suppression, gating, tracking, and ITV techniques, are all recommended.

Localization using daily image guidance device

This study requires IGRT. CBCT, MVCT, dual fixed position in room kV imaging system, and in-room diagnostic CT are all accepted. Simple portal images are not suitable for this study.

**4. Target Volume**

Since the number and distribution of recurrent ovarian cancer lesions are distinctly different from those of other solid cancers, the method to determine the number is based on the concept of anatomical region. In other words, plural lesions can be counted as single lesion within the anatomical region (bold) defined as below to be included in single radiation plan. For these caese, ten fractions are recommended.

Definition of lesion site

*Cervical lymph nodes*: divided by left and right. Level 1-6 lymphatic and retropharyngeal areas are also included.

- Left cervical lymph node

- Right cervical lymph node

*Lung:* divided by left and right. classified in detail as follows.

- Center of the left lung, periphery of the left lung, and area of the left thoracic membrane

- Center of the right lung, periphery of the right lung, and area of the right thoracic membrane

*Central lung:* It is defined as a GTV located within 2 cm of the proximal bronchi as described in RTOG 0813/0915.

*Lung periphery:* Tumors that do not belong to the central lung of the lung parenchyma

*Pleural:* includes seeding tumors on the pleura separated from the lung parenchyma

*Mediastinal lymph nodes*: from the superior part of sternums to the diaphragm. Sternal metastases are included in the mediastinal/neck lymph nodes

*Liver and spleen:* tumors in the hepatic parenchyma and adjacent rib metastases also belong to this area. It is divided into the left and right lobe.

- the left lobe of the liver

- the right lobe of the liver

- Perihepatic space; recurrent tumor in the space between hepatic parenchyma and capsule

- Spleen

- Perisplenic space; recurrent tumor of space between spleen parenchyma and capsule

*Vertebrae*: Tumors located within 1 cm around vertebral body are included in the vertebrae. Separate lesions within the upper or lower 2 vertebrae is calculated as single lesion. Any spinal tumors fall into one of the three types below.

*Non-vertebral bones*:

- Tumor located within 1 cm around vertebral body is considered spinal metastasis.
- Lesions on rib or scapula adjacent to the lung within the thoracic wall are classified as lung metastases.
- Osseous metastases adjacent to the mediastinum and neck structures are classified as mediastinal/cervical lymph nodes.
- Rib metastases located within 1 cm around the liver are classified as a hepatic region.
- Rib metastases close to the stomach and abdominal wall are classified as an abdominal cavity region.
- Metastases on the sternal region are classified as mediastinal/cervical lymph nodes.

*Abdomen:* Refers from diaphragm to the pelvic inlet (the level of common iliac lymph node) and excludes liver/spleen area. Classified as follows

*-* abdominal cavity

*-* paraaortic lymph node; refers to lymph nodes from common iliac vessels to the diaphragm. The lymph nodes in this area are calculated as single lesion.

*pelvis:* Refers from the pelvic inlet to the anus.

-pelvic

-pelvic lymph node; Refers from bifurcation of common iliac lymph node to obturator lymph node

Definition of treatment volume by metastatic site

GTV includes all metastatic sites observed by planning CT, additional PET/CT, or MRI.

The PTV margin is 3-5 mm. Also, If the boundary between the PTV and the normal organ is unclear, set 2 mm extension from the outer edge of the normal organ as the boundary of the PTV. For spinal tumors, PTV is determined according to the target determination guideline of RTOG 0631, as shown in the figure below.

**5. Treatment Plan**

General considerations

The prescription isodose curve covering 95% of the PTV can be set at 60-100% with the maximum dose of 100%. A hotspot should be present only within the PTV.

(e.g., 30 Gy/ 0.6 = 50 Gy when 30 Gy is prescribed for a 60% isodose curve)


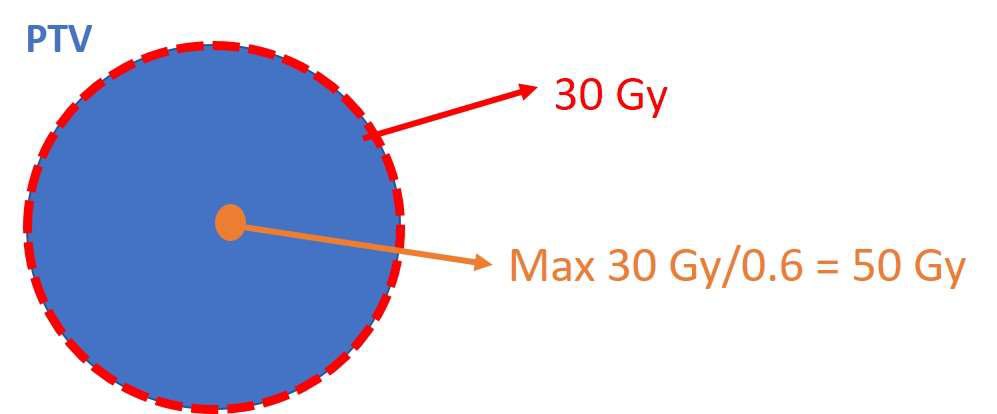


NOTE:

1. Normalization: The treatment plan is normalized at first so that 100% corresponds to the maximum dose in the PTV (MAXPTV). This point corresponds to the PTV center of the tumor and may be located elsewhere within the PTV.
2. Prescription Isodose Surface Coverage: The prescription isodose surface is selected to include 90% of the PTV in the prescription isodose curve. Doses less than 90% of the prescription dose are limited to the outer boundary of the PTV. (Exceptionally, 70% coverage is allowed for some PTVs if small lesions are collectively formed and have irregular shapes. However, in any case, maximum efforts should be made to ensure that GTV covered by a 100% prescription isodose surface). The prescription isodose surface should be determined from 60% to 100% of the maximum dose in the PTV (MAXPTV). (Maximum dose should be 100-166.67% based on prescription isodose surface). MAXPTV corresponds to the normalization point (100%).
3. Target dose heterogeneity: SABR should prioritize proper minimum target inclusion and rapid dose fall-off gradient outside the target rather than target dose uniformity. Hotspots within the target are acceptable as they are usually generated in the tumor. The only exception is when a hotspot in the PTV crosses the OAR.
4. OAR protection and PTV_EVAL setting; If considerable volume of OARs are included in PTV, treatment plan using 10 fx is recommended, and in this case, PTV_EVAL separately can be set to evaluate PTV coverage.
5. PTV_EVAL is defined as the remaining part of pre-defined PTVs excluding OARs (bowel, duodenum, rectum, bladder, etc.) during treatment planning.
6. However, in this case, even if the evaluation of target is based on PTV_EVAL, minimum dose of PTV should be 70% or more of the prescribed dose.
7. In this case, if it is difficult to achieve R_50%_ conditions excluding the evaluation of the ratio of the prescription isodose volume, it is evaluated as an acceptable treatment plan even if it is not met. (However, evaluations of R_50%_ and D_2cm_ are evaluated using the existing PTV volume, and lowering OAR dose should be prioritized rather than dose fall-off in this case).
8. When treatment plan with PTV_EVAL is evaluated, if necessary, prior consultation should be communicated to the research headquarter to prevent from being evaluated as an unacceptable deviation
9. A single plan with multiple targets; when single treatment plan covers multiple targets, evaluation of the treatment plan should be aimed at a single treatment plan summing up all targets, not individual targets.
10. High-Dose Spillage
11. Location: Doses greater than 110% of the prescription dose should be within the PTV not in normal tissue outside the PTV. Up to 120% coverage is allowed for PTVs if small lesions are collectively formed and have irregular shapes. In other words, there should be no dose greater than 120% of the prescription dose at any point outside the PTV. In any case, maximum effort should also be made to maintain the ratio of the prescription isodose volume (V100/VPTV) to the PTV below at 1.2-1.5.
12. Volume: Acceptable isodose distributions should be as conformal as possible. The smaller the ratio of the prescription isodose volume to the PTV, the better.
13. The ratio of the prescription isodose volume to the PTV should be less than 1.2. The acceptable ratio is 1.2 to 1.5. A ratio of 1.5 or more is considered an unacceptable violation. A prescription line for each lesion is drawn for calculating this ratio.
14. The following table shows the guidelines for the ratio of the 50% prescription isodose volume to the PTV (R_50%_) and the maximum dose at a point of 2 cm from the PTV. As it is difficult to limit the 50% isodose volume when aggregating the treatment dose for multiple metastases, this ratio should be evaluated for dose determination for a single metastatic lesion at a time.

| **PTV Volume (cc)** | **Ratio of 50% Prescription Isodose Volume to PTV**  **Volume, R_50%_** | **Maximum Dose at 2cm (D_2cm_) from PTV in any direction as %**  **of Prescribed Dose** |
| --- | --- | --- |
| 1.8 | < 7.5 | <57.0 |
| 3.8 | < 6.5 | <57.0 |
| 7.4 | < 6.0 | <58.0 |
| 13.2 | < 5.8 | <58.0 |
| 22.0 | < 5.5 | <63.0 |
| 34.0 | < 5.3 | <68.0 |
| 50.0 | < 5.0 | <77.0 |
| 70.0 | < 4.8 | <86.0 |
| 95.0 | < 4.4 | <89.0 |
| 126.0 | < 4.0 | <91.0 |
| 163.0 | < 3.7 | <94.0 |

NOTE: If the PTV volume or length is not shown above, it is calculated by linear interpolation between sections.

NOTE: Lesions within 2 cm of the skin hardly meet the D_2cm_ and R_50%_ values. In this case, these criteria are not applied.

NOTE: When small lesions are collectively formed and PTVs has an irregular shape, each plan is not available for each target because of proximity, and D_2cm_ criterion is not applied. R_50%_ is another evaluation item whether to achieve rapid fall-off of SABR. The values presented in the table above should be applied, but if not achieved, the review process should be used to determine whether to include clinical trial.

1. If considerable volume of OARs is included in PTV and high possibility of toxicity is expected, “PTV_EVAL” separately can be set to evaluate PTV coverage at the discretion of physician and the indications and details are as follows. In this case, treatment plan using 10 fx is recommended.
2. Indication of PTV_EVAL use.

- Bowel toxicity is highly expected due to close proximity to PTV (e.g. treatment for peritoneal or pelvic seeding lesions)
- When re-SABR is performed on previously treated lesion with SABR after registration of the study
- When toxicity is highly expected by the judgment of a radiologic oncologist (e.g. large PTV volume, etc.)

1. PTV_EVAL is defined as the remaining part of pre-defined PTVs excluding OARs (bowel, duodenum, rectum, bladder, etc.) during treatment planning
2. However, in this case, even if the evaluation of target is based on PTV_EVAL, minimum dose of PTV should be 70% or more of the prescribed dose.
3. R_50%_ and D_2cm_ (see table below) are evaluated using "PTV", but in this case, they are evaluated as acceptable treatment plans even if the conditions are not met.
4. When treatment plan with PTV_EVAL is evaluated, if necessary, prior consultation should be communicated to the research headquarter to prevent from being evaluated as an unacceptable deviation.

Priority in Treatment Planning

Every effort should be made to fully satisfy all treatment goals and normal organs (OAR), otherwise it will be determined as an unacceptable error. There is a margin of error as it may not possible to meet all the best standards in some cases. Therefore, priorities of the treatment goals are suggested as follows:

1. Dose limitation in the spinal cord, cauda equina, sacral and brachial plexus
2. Dose “compactness” conditions should be met, including prescription isodose surface coverage, high-dose spillage (location and volume) and moderate-dose spillage (D_2cm_ and R_50%_). This is because these indicators define the goal of the SABR. The dose compactness should be evaluated for treatment plans based on a single lesion. Dose limitation for normal organs should be met based on an integrated treatment plan.
3. Dose limiting conditions for critical structures other than those listed in Listing 1 should be met. The dose limiting condition for normal organs has the lowest priority (except for the allowed dose for the nervous system) as it is less validated. The goals of the SABR plan are mainly captured by the dose compactness criteria, which justifies a higher priority. If all goals cannot be achieved, for example, achieving the target dose compactness without deviations is allowed. Moreover, it is also allowed to sacrifice non-spinal, normal tissue with acceptable deviations. Unacceptable deviations should be avoided in all cases.
4. Only in the cases where PTV coverage cannot be achieved while avoiding unacceptable deviations for normal organs, a fraction of the PTV can be included (see Section 5.2.6). Or, when it is directly adjacent to normal organs, the PTV coverage may be low as 70% of the prescription dose. Upon completion of this process, the research team should be contacted to ensure that the result is not evaluated as unacceptable deviation.

**6. Critical Structures**

Note: When submitting the RT data, all critical structures should be labeled as listed in a Table below.

| ***Standard Name*** | ***Description*** |
| --- | --- |
| PTV_2000, PTV_3000, PTV_3500, or  PTV_4000 |  |
| GTV_2000, GTV_3000, GTV_3500, or  GTV_4000 |  |
| BrachialPlex_L | Left Brachial Plexus |
| BrachialPlex_R | Right Brachial Plexus |
| Trachea | Trachea |
| BronchialTree | carina, right and left main bronchi, right and left upper lobe bronchi, intermedius bronchus, right middle lobe bronchus, lingular  bronchus right and left lower lobe bronchi |
| BronchTree_20 | Proximal bronchial tree expanded by 2cm |
| Larynx | Larynx |
| Esophagus | Esophagus |
| GreatVessels | Great Vessels |
| ChestWall | Chest wall |
| Rib | Ribs within 5 cm of the PTV should be contoured |
| Heart | Heart |
| Lungs | Combined Left and Right Lungs |
| Lung_R | Right Lung |
| Lung_L | Left Lung |
| Stomach | Stomach |
| Liver | Liver |
| BileDuct | Bile duct |
| Kidneys | Combined Left and Right Kidneys |
| Kidney_R | Right Kidney |
| Kidney_L | Left Kidney |
| Ureter | Ureter |
| Femurs | Both Femurs |
| Duodenum | Duodenum |
| Bladder | Bladder |
| Bowel | Large and Small Bowel |
| SpinalCord | Spinal cord |
| CaudaEquina | Cauda equine |
| SacralPlexus | Sacral plexus |
| Rectum | Rectum |
| External | Body surface |
| Skin | skin will be defined as the outer 0.5 cm of the body surface |

*Spinal cord*

The spinal cord should be contoured based on a osseous boundary of the spinal canal that ends at L2. Also, the spinal cord should be contoured from at least 10 cm above the upper boundary of the PTV, continuous on all cross-sections of the CT, and contoured to 10 cm below the lower boundary of the PTV.

*Cauda equina*

The site should start from the conus medullaris (the tip of the spinal cord, usually near L1 or L2) to include all the spinal canals, sacral vertebrae, and filum terminale.

*Sacral plexus*

It should include the nerve roots from L5 to S3 on both sides, ranging from the vertebral neuropore to the obturator internus, where the nerve coalesces.

*Esophagus*

The esophagus should be contoured using the mediastinal window of CT to include the mucosa, submucosa, lumen, and all muscle layers to the adipose adventitia. Also, it should be contoured from at least 10 cm above the upper boundary of the PTV, continuous on all cross-sections of the CT, and contoured to 10 cm below the lower boundary of the PTV.

*Brachial plexus*

Ipsilateral brachial plexus is defined as the region starting from where the nerve passes the spinal neuropore of C5 to T2 on the ipsilateral side. Given the purpose of this study, however, only the main branches of the brachial plexus identified using the subclavicular and midaxillary blood vessels are contoured. This neurovascular complex should be contoured along the axillary vein, starting from the brachial bifurcation following the subclavicular vein to where the neurovascular complex passes through the second rib. If the PTV of all metastatic lesions is located 10 cm or farther away from the brachial plexus, contouring of this structure is not required.

*Heart*

The heart should be contoured along the pericardium. Considering the purpose of contouring, the upper boundary should be contoured from the lower part of the aortic arch (aortopulmonary window) to the apex of the heart.

*Respiratory tract and proximal bronchi*

The respiratory tract and proximal bronchi should be contoured as two separate structures. Using the mediastinal window of CT, the respiratory tract related to the structures such as the mucosa, submucosa, tracheal ring, etc. is contoured. To do this, the organ is divided into two parts: proximal bronchi and distal 2 cm bronchi. The proximal bronchi are contoured as a single structure, while the distal 2 cm bronchi should be included in the structure identified as the proximal bronchi.

- Proximal respiratory tract

In the case of lung metastases, the proximal respiratory tract should be contoured at least 10 cm above the upper boundary of the PTV or 5 cm above the trachea bifurcation (whichever is located upper), descending to the upper boundary of the proximal bronchi.

- Proximal bronchi

As shown in Figure 5-1, the contouring of the proximal bronchi includes the lowest 2 cm of the distal respiratory tract and the proximal respiratory tract on both sides. The following respiratory tract is included depending on the standard anatomical relationship: the distal 2 cm of trachea, carina, right and left main bronchi, left and right upper lobe bronchus, middle bronchus, right middle bronchus, lingular bronchus, left and right lower lobe bronchus. The contouring of the lobar bronchus ends at the segmental bifurcation. If a part of the proximal bronchi is within the GTV, it should be contoured as “proximal bronchi GTV” not as “proximal bronchi."

*Whole lung*

Both the left and right lungs should be contoured as a single structure. The contouring is performed using the lung window. Any lungs inflated and collapsed should be contoured as follows: however, the tumor (GTV) and trachea/ipsilateral bronchi should not be included in this structure as defined.

*Proximal bronchi plus 2 cm*

One of the methods for determining whether a lung metastasis is central or peripheral is to see whether it is in the proximal bronchial region. The RTOG protocol for the SABR has defined an artificial organ larger than 2 cm in all directions from the proximal bronchi. If the GTV is in this structure, the patient is suitable for this protocol. Most treatment plan systems have an automatic contouring function for creating this structure, which facilitates the establishment of the treatment plan. This structure is not required by the protocol. However, contouring is recommended to select suitable patients. Alternatively, a graduated ruler may be used on the treatment plat to ensure that it is suitable for the protocol.

*Skin*

The outline of the patient is contoured. A normal organ of the skin is defined as 0.5 cm outer of the body surface. In other words, it is a uniform thickness (0.5 cm) covering the whole body on the transverse plane. The surfaces of the upper and lower boundaries of the planned CT are not contoured unless the skin is actually in this position (e.g., the scalp on top of the head).

*Great vessels*

Great vessels (aorta and vena cava, not pulmonary artery or vein) should be contoured along the vessel wall by using a mediastinum window on a CT scanner and contoured all the way to the adipose adventitia. Also, it should be contoured starting from at least 10 cm above the upper boundary of the PTV, continuous on all cross-sections of the CT, and to 10 cm below the lower boundary of the PTV. For tumors located on the right, the vena cava should be contoured, while the aorta should be contoured for those on the left.

*Cases not adjacent to the wall of the structure*

For esophagus, trachea, proximal bronchi, and great vessels, the non-adjacent wall is the half the circumference of the tubular structure that is not in direct contract with the GTV or PTV. The contouring starts at the top of the indicated structure and ends at the bottom. Half of the lumen of the structure should be included in this contour.

*Stomach*

The entire stomach and its contents should be contoured as a single structure from the esophagus to the first segment of the duodenum.

*Duodenum*

From the end of the stomach, the walls and contents of the first, second, and third segments of the duodenum should be contoured as a single structure. Also, it should be contoured until the superior mesenteric artery passes through the third segment of the duodenum.

*Intestine (large/small)*

The intestine should be contoured to include the ileocecal valve, ascending, transverse, descending, and sigmoid colons as a single structure.

*Rectum*

The entire rectum should be contoured with its contents from the peritoneal reflection of the sigmoid colon to the anus.

*Urinary bladder*

Only the wall of the urinary bladder should be contoured, excluding urine.

*Kidney (renal cortex)*

Both the right and left kidneys should be contoured (renal cortex) except for the renal pelvis/collecting system.

*Liver*

The entire liver should be contoured except for the GTV.

*Bile duct*

To contour the outline of the bile duct, the area from the site where the portal vein joins the splenic vein to the site where the liver is divided into the right and left is used as a marker to identify the bile duct.

*Femoral head*

The femoral head and the articulations of the sphenoid should be contoured.

*Rib*

The ribs within 5 cm of the PTV should be contoured with the outline of the cortical bone, including the marrow cavity. In general, several parts of adjacent ribs should be contoured as a single structure. However, the adjacent ribs should not be contoured continuously (i.e., the intercostal space is not included as a part of the ribs).

*Other structures*

The dose limit table above contains other structures. It is required if a structure is within 3 cm of the PTV.

*Dose-volume limit to major organs*

Complex dose distribution in normal organs is important to understand the toxicity after the SABR. To evaluate protocol compliance, a combined dose plan, including all treated metastases and normal organs, and an individual SABR plan should be submitted. To facilitate the combined plan, doses for all metastases should be calculated simultaneously in a single CT scan with a resolution of at least 2 x 2 x 3 mm. If not possible, a combined plan should be established by integrating the dose for each metastatic lesion treated. The combined dose of major normal organs is used to evaluate protocol compliance.

Note: If a point in the OAR (0.03 cc) has a total dose contribution of > 5 Gy for a complex plan of several treatment sites, it is recommended to use the same dose to treat all sites.

Note: If different dose plans are used in a given patient, the dose limit to normal organs receiving > 1 Gy contribution in individual metastasis treatment should be evaluated according to the table with the smallest number of treatment segments (e.g.,

If a patient is treated by the plan with one and five treatments, the plan with 1 treatment is used to evaluate the limit of normal organs.

Priorities of the plan on normal organs:

Dose to the spinal cord is an absolute limit, and exceeding this limit constitutes an unacceptable violation. However, some normal organs (i.e., esophagus, trachea, bronchi, and heart in the lungs) may be located adjacent to the GTV/PTV to be treated. Therefore, there are no specific limitations to tumors in direct contact with such organs, as they cannot be treated with the prescription dose without irradiating the organs with the prescription dose. In such cases where the corresponding organ is a part of the GTV/PTV, it should be planned that there are no hotspots in the organ with a dose of greater than 105% of the prescription dose (unacceptable violation). (If small lesions are collectively formed and the PTV has an irregular shape, 120% can be allowed if difficult to meet the 105% criterion. If the 120% standard is applied, maximum effort should also be made to maintain the ratio of the prescription isodose volume (V100/VPTV) to the PTV below at 1.2-1.5.) In addition, the volume of the corresponding normal organ should be minimized in terms of length and width (i.e., circumference). Efforts should be made to reduce the dose to the contralateral wall of the organ. As for parallel normal organs, if the dose exceeds 110% or greater of the limit dose, it is considered an unacceptable violation.

In the case of non-spinal organs with known sensitivity to high-dose radiation (including the intestine, esophagus, and stomach) contained within the PTV or immediately adjacent to the PTV, the prescription dose should be used at the lower limit of acceptable variation. Also, every effort should be made to cover the GTV with the prescription dose while ensuring a rapid dose decrease for normal organs. The applicable range for sections containing normal organs or immediately adjacent to the PTV may be as low as 70% of the prescription dose. Every effort should be made to cover 100% of the GTV with the prescribed dose at the lower limit of the acceptable variation. As tumors and normal organs may not be strictly avoided, if a larger volume limit is exceeded, it is not scored as an unacceptable variation.

For tumors that are not in direct contact with normal organs, the study sites are encouraged to comply with the principles of a careful treatment plan to avoid unnecessary radiation exposure to major normal structures. We expect normal organ doses to be as low as achievable (ideally, < 6 Gy/fraction).

**7. Dose-Volume Limit to Normal Organs**

The normal organ dose in the table below is based on the NRG-LU002 trial and SABR-COMET-10 study. If the normal organ dose is not presented in the table below, the alpha-beta ratio of the late toxicity is set to 2, and the QUANTEC dose is followed by calculating the dose with the linear-quadratic formula.

**Dose Constraints for Serial Structures.**

D0.03cc = maximum dose in Gy allowable to the hottest 0.03 cc; other D values are used in the same way

| **Structure** | **Volume** | **1 Fraction** | **3 Fraction** | **5 Fraction** |
| --- | --- | --- | --- | --- |
| **Spinal Cord** | D0.03cc | 14 | 22.5 | 28 |
|  | D0.35cc | 10 | 15.9 | 22 |
| **Cauda Equina or Sacral Plexus** | D0.03cc | 16 | 22.5 | 31.5 |
|  | D5cc | 14 | 21.9 | 30 |
| **Esophagus** | D0.03cc | 15.4 | 25.2 | 35 |
|  | D5cc | 11.9 | 17.7 | 19.5 |
| **Brachial Plexus** | D0.03cc | 16.4 | 26 | 32.5 |
|  | D3cc | 13.6 | 22 | 27 |
| **Heart** | D0.03cc | 22 | 30 | 38 |
|  | D15cc | 16 | 24 | 32 |
| **Great Vessels** | D0.03cc | 37 | 45 | 53 |
|  | D10cc | 31 | 39 | 47 |
| **Trachea and large bronchi (mainstem, bronchus intermedius)** | D0.03cc | 20.2 | 30 | 40 |
|  | D4cc | 17.4 | --- | --- |
|  | D5cc | --- | 25.8 | 32 |
| **Chest Wall or Rib** | D0.03cc | 33 | 50 | 57 |
|  | D5cc | 28 | 40 | 45 |
| **Skin** | D0.03cc | 27.5 | 33 | 38.5 |
|  | D10cc | 25.5 | 31 | 36.5 |
| **Stomach** | D0.03cc | 22 | 30 | 35 |
|  | D10cc | 17.4 | 22.5 | 26.5 |
| **Bile Duct** | D0.03cc | 30 | 36 | 41 |
| **Duodenum** | D0.03cc | 17 | 22.2 | 26 |
|  | D5cc | 11.2 | 15.6 | 18.5 |
|  | D10cc | 9 | 12.9 | 14.5 |
| **Jejunum or Ileum** | D0.03cc | 22 | 27 | 32 |
|  | D30cc | 12.5 | 17.4 | 20 |
| **Colon** | D0.03cc | 29.2 | 34.5 | 40 |
|  | D20cc | 18 | 24 | 28.5 |
| **Rectum** | D0.03cc | 44.2 | 49.5 | 55 |
|  | D3.5cc | 39 | 45 | 50 |
|  | D20cc | 22 | 27.5 | 32.5 |
| **Ureter** | D0.03cc | 35 | 40 | 45 |
| **Bladder** | D0.03cc | 25 | 33 | 38 |
|  | D15cc | 12 | 17 | 20 |
| **Penile Bulb** | D3cc | 16 | 25 | 30 |
| **Femoral Heads** | D10cc | 15 | 24 | 30 |

**Dose Constraints for Parallel Structures**.

Parallel structures require the use of a ‘critical volume’ (CV), also termed a ‘complementary volume’. For example, for lung, the CV1500cc is listed as 7 Gy for 1-fraction, meaning that there must be 1500 cc of lung receiving 7 Gy or less. This is read from the left-hand side of a DVH. VX refers to the percent of lung (minus GTVs) receiving X Gy or more.

| **Structure** | **Volume** | **1 Fraction** | **3 Fraction** | **5 Fraction** |
| --- | --- | --- | --- | --- |
| **Lung (combined right and left, subtract GTVs)** | CV1500cc | 7 | 10.5 | 12.5 |
|  | V8Gy(%) | 37 |  |  |
|  | V11Gy(%) |  | 37 |  |
|  | V13.5Gy(%) |  |  | 37 |
| **Liver** | CV700cc | 11 | 17.1 | 21 |
| **Kidney cortex (combined left**  **and right)** | CV200cc | 9.5 | 15 | 18 |

CV is comprised of three steps as in the example below. First, find and read the threshold dose for CV in the DVH curve. At the DVH in the figure below, 1200 cm^3^ of the lung receives ≥12.5 Gy. Second, it can be seen that the total lung volume is 3600 cm^3^ from the y-intercept of the curve. Lastly, it can be seen that the volume of the lung that received less than the threshold dose is 2400 cm^3^ (3600-1200 cm^3^). As it is greater than 1500 cm^3^, the standard, it can be said that this treatment plan meets the dose-limiting condition.

| **Serial tissue** | **Volume** | **Volume**  **max (Gy)** | **Max point**  **dose (Gy)** | **Endpoint**  **(grade ≥3)** |
| --- | --- | --- | --- | --- |
| Optic pathway | <0.5 cm3 | 30.6 | 33.1 | Neuritis |
| Eye (retina) | Mean dose | <26 | 30 | Retinitis |
| Lens |  |  | 7 | Cataract |
| Eyelid, meibomian glands  (one side) |  |  | 21.3 | Dry eye  syndrome |
| Lacrimal gland (one side) | <1 cm3 | 14.1 | 23.6 | Lack of tears |
| Cochlea | <0.5 cm3 | 25 | 27 | Hearing loss |
| Brain stem (not medulla) | <5 cm3 | 32 | 38 | Cranial  neuropathy |
| Spinal cord | <5 cm3 | 31 | 36 | Myelitis |
| Salivary gland (one side) | <7 cm3 | 14.1 | 21.3 | Xerostomia |
|  | mean dose | <17.7 |  |  |
| Larynx | <3 cm3 | 30 | 45 | Necrosis/edema |
| TM joint | <1 cm3 | 37.7 | 414.4 | Inflammation |
| Cauda equina | <5 cm3 | 35 | 41 | Neuritis |
| Sacral plexus | <5 cm3 | 35 | 41 | Neuropathy |
| Esophagus | <5 cm3 | 40 | 48 | Esophagitis |
| Brachial plexus | <3 cm3 | 37 | 43 | Neuropathy |
| Heart/pericardium | <15 cm3 | 36.6 | 42.5 | Pericarditis |
| Great vessels | <10 cm3 | 55.7 | 62.9 | Aneurysm |
| Trachea and large bronchus | <5 cm3 | 52 | 59 | Impairment of pulmonary  toilet |
| Skin | <10 cm3 | 46.3 | 48.9 | Ulceration |
| Stomach | <50 cm3 | 33.9 | 45 | Ulceration/fistula |
| Duodenum | <5 cm3 | 33.9 | 45 | Ulceration |
| Jejunum/ileum | <120 cm3 | 33.9 | 41 | Enteritis/  obstruction |
| Renal hilum/vascular trunk | 15 cm3 | 30.7 |  | Malignant  hypertension |
| Colon | <20 cm3 | 47 | 60 | Colitis/fistula |
| Rectum (including stool) | <10 cm3 | 52 | 65 | Proctitis/fistula |
|  | <20 cm3 | 49 |  |  |
|  | <30 cm3 | 46 |  |  |
|  | <40 cm3 | 43 |  |  |
| Bladder (with urine) | <90 cm3 | 48 | 53 | Cystitis/fistula |
|  | <120 cm3 | 45 |  |  |
| Bladder (suprapubic wall) | <5 cm3 | 23 | 42 | Dysuria |
| Penile bulb | <3 cm3 | 38 | 44 | Erectile  dysfunction |
| Femoral heads | <10 cm3 | 38 | 43.5 | Necrosis |
| **Parallel tissue** | **Critical volume (cm3)** | **Critical volume dose max**  **(Gy)** | **Other constraints** | **Endpoint (grade ≥3)** |
| Lung (right and left) minus  GTV | 1500 for  males | 15 |  | Basic lung  function |
|  | and 950 for  females* |  |  |  |
| Lung (right and left) minus  GTV |  |  | V-16 Gy  <37% | Pneumonitis |
| Liver minus GTV | 700 cm3* | 27 |  | Basic liver  function |
| Renal cortex (right and  left) | <200 cm3 | 21 |  | Basic renal  function |

* One-third of the “native” total organ volume (before any resection or volume reducing disease), whichever is greater.

Abbreviations: CT = computed tomography; GE = gastroesophageal; GTV = gross target volume; PTV = planning target volume; TM = temporomandibular

Dose limit to the Rib/thoracic wall

The ribs and thoracic wall close to the treated lesion have a risk of complications. The location of a tumor, particularly if it is located peripheral, may increase the potential risk of thoracic wall toxicity. The target coverage should not be compromised to limit the rib/thoracic wall dose, and every effort should be made to minimize the dose on normal organs.

**8. Compliance Criteria**

Duration of treatment

The duration of treatment is defined for each metastatic lesion.

*Per protocol*

Single fraction radiotherapy: The SABR should be completed within six weeks of randomization/enrollment.

Three-fraction radiotherapy: All 3 fractions of the SABR should be completed within three weeks of the first dose of SABR and within six weeks of the randomization/enrollment.

Five-fraction radiotherapy: All 5 fractions of the SABR should be completed within three weeks of the first dose of SABR and within six weeks of the randomization/enrollment.

*Acceptable Variation:* treatment completion > 3 weeks - < 4 weeks

*Unacceptable Deviation:* treatment completion > 4 weeks

Compliance with the PTV dosimetry regulations

The prescription dose outside of the acceptable dose of the protocol prescription dose (the dose accounting for 95% of the PTV) is scored as an unacceptable deviation. If the score in the PTV range is 9% or more, the case is acceptable, acceptable variation for 70-95%, and unacceptable deviation for < 70%.

Compliance with the normal organ dosimetry

The dose of a critical structure should be based on the complex dose distribution when at least one compliance is treated. Dose limits to the spinal, caudal, sacral, and brachial plexus doses are respected. Any dose to the spinal cord, caudal and sacral plexus higher than those listed in the table is considered an unacceptable deviation. If all other normal organs, doses irradiated to serial normal organs greater than or equal to 105% of the dose prescribed for the PTV are scored as unacceptable deviations when the dose criteria for normal organs cannot be met. A parallel normal organ dose greater than 110% of the dose prescribed for the PTV is scored as an unacceptable deviation. Doses in the range between the numbers in the table and unacceptable deviations are considered acceptable variations.

**9. Details of Selected Cases**

In the SABR-ROC study, there are no restrictions regarding the treatment sites for recurrent ovarian cancer lesions, allowing for the contemplation of various therapeutic scenarios across diverse cases. Consequently, four patient cases have been selected, each representing different anatomical sites treated with distinct approaches.

Patient Case 1 involves a diagnosis of high-grade serous ovarian carcinoma with metastasis to the left supraclavicular lymph nodes. The patient initially received three cycles of neoadjuvant chemotherapy (paclitaxel and carboplatin), followed by debulking surgery, pelvic and supraclavicular lymph node resection, and Hyperthermic Intraperitoneal Chemotherapy (HIPEC) in January 2018. Subsequent adjuvant chemotherapy was administered through six cycles until June 2018. Eight months post-primary treatment, the patient experienced a recurrence in the retroperitoneal lymph nodes, necessitating a second-line chemotherapy regimen (Belotecan and cisplatin), which was completed up to five cycles between February and May 2019, interrupted by sepsis. Nine months after completing the second-line chemotherapy, another recurrence at the retroperitoneal lymph nodes led to a third-line treatment with six cycles of pegylated liposomal doxorubicin and carboplatin from March to August 2020. A PET-CT scan for response evaluation showed partial remission of existing lesions but revealed new lesions nearby. The patient was referred for salvage radiotherapy on the retroperitoneal lymph nodes and is scheduled to start Niraparib.

Patient Case 2 was diagnosed with serous papillary adenocarcinoma of the ovary and underwent initial debulking surgery, pelvic lymph node resection, and mesenteric resection in September 2007, followed by six cycles of adjuvant paclitaxel and carboplatin until December 2007. After four years and seven months, a mesenteric recurrence was treated with adhesiolysis and another six cycles of paclitaxel and carboplatin from August to November 2012. A subsequent recurrence led to small bowel resection and a third round of chemotherapy (belotecan and cisplatin) for six cycles from August to December 2014. Two years later, metastases to the supraclavicular, mediastinal lymph nodes, and lungs required a fourth chemotherapy course (paclitaxel and carboplatin) for six cycles from January to April 2017. Nine months later, recurrences in the peritoneal cavity and around the aorta were treated with a fourth-line regimen (liposomal doxorubicin and carboplatin) from May to September 2018. After 20 months, multiple pulmonary metastases and intraperitoneal spread led to tumor debulking and right hemicolectomy in September 2020, followed by seven cycles of adjuvant paclitaxel and carboplatin from September 2020 to February 2021. The patient was then referred for radiotherapy for residual lung metastases.

Patient Case 3 was diagnosed with papillary adenocarcinoma of the ovary and underwent initial debulking surgery, pelvic lymph node resection, and partial mesenteric resection in November 2011. Post-surgery, the patient received six cycles of adjuvant paclitaxel and carboplatin from December 2011 to March 2012. Recurrent peritoneal metastases near the liver occurred three years and one month later, and again after two years and three months, leading to further chemotherapy: six cycles of paclitaxel and carboplatin until August 2015, followed by liposomal doxorubicin and carboplatin until June 2018. In April 2022, after three years and ten months, the patient was referred for salvage radiotherapy for regrowing seeding metastasis around the liver. Due to advanced age and patient refusal, no further chemotherapy was planned.

Patient Case 4 was diagnosed with high-grade serous carcinoma of the ovary with metastasis to the left supraclavicular lymph nodes. The initial treatment included three cycles of neoadjuvant chemotherapy (paclitaxel and carboplatin), debulking surgery, resection of pelvic and aortic lymph nodes, and mesenteric resection in November 2014. This was followed by six cycles of adjuvant chemotherapy completed by April 2015. Three years and six months later, the patient experienced a submucosal recurrence in the stomach and sigmoid colon, leading to six cycles of second-line chemotherapy (paclitaxel, carboplatin, and bevacizumab) completed by May 2019. In September 2020, a recurrent gastric lesion was addressed with laparoscopic wedge resection. Ten months later, recurrences in the peritoneum and subcarinal lymph node necessitated a third round of chemotherapy (liposomal doxorubicin and carboplatin) for six cycles completed by December 2021. Eleven months later, an intraperitoneal recurrence occurred, and the patient was referred for salvage radiotherapy.

**Supplementary Table 1. Submission Status of Targets and Plans During Dummy Runs Across Participating Centers**

| **Dummy Run** | **Participating center** | | | | | | | | | | | | | |
| --- | --- | --- | --- | --- | --- | --- | --- | --- | --- | --- | --- | --- | --- | --- |
|  | **A** | **B** | **C** | **D** | **E** | **F** | **G** | **H** | **I** | **J** | **K** | **L** | **M (headquarter)** |  |
| Target | ○ | × | ○ | ○ | ○ | ○ | ○ | ○ | × | ○ | ○ | ○ | ○ |  |
| Plan | ○ | ○ | ○ | × | ○ | ○ | × | ○ | ○ | × | ○ | ○ | ○ |  |

A: Asan Medical Center, B: Chung-Ang University Hospital, C: Seoul St. Mary's Hospital, D: St. Vincent's Hospital, E: Dong-a University Hospital, F: Keimyung University Dongsan Medical Center, G: Ewha Womans University Medical Center, H: Gangnam Severance Hospital, I: Gil Medical Center, J: Korea University Medical Center, K: Samsung Medical Center, L: Seoul National University Bundang Hospital, M: Yonsei Cancer Center

**Supplementary Table 2. Comparison of Compliance with PTV Dosimetry Regulations for Each Case Without Considering Multiple Target Volume for Single Plan**

| **Case** | **Features** | | **Participating center** | | | | | | | | | |
| --- | --- | --- | --- | --- | --- | --- | --- | --- | --- | --- | --- | --- |
|  |  |  | **A** | **B** | **C** | **E** | **F** | **H** | **I**^*^ | **K**^*^ | **L**^†^ | **M**^†^ |
| Case 1 | PTV1^‡^ | Deviations | 1 | 2 | 1 | 1 | 1 | 1 | 1 | 1 | 2 | 2 |
|  |  | Parameters | R_50%_ | PTV coverage R_50%_ | R_50%_ | R_50%_ | R_50%_ | R_50%_ | R_50%_ | R_50%_ | PTV coverage R_50%_ | PTV coverage R_50%_ |
| Case 2 | PTV1 | Deviations | 0 | 3 | 2 | 3 | 0 | 2 | 2 | 1 | 2 | 0 |
|  |  | Parameters | - | PTV coverage R_50%_ D_2cm_ | R_50%_ (major) D_2cm_ (major) | High-dose Spillage R_50%_ D_2cm_ | - | R_50%_ D_2cm_ | R_50%_ D_2cm_ | R_50%_ | PTV coverage R_50%_ | - |
|  | PTV2^‡^ | Deviations | 2 | 3 | 2 | 2 | 1 | 2 | 2 | 2 | 2 | 1 |
|  |  | Parameters | R_50%_ D_2cm_ | PTV coverage R_50%_ D_2cm_ | R_50%_ D_2cm_ | R_50%_ D_2cm_ | R_50%_ | R_50%_ D_2cm_ | R_50%_ D_2cm_ | R_50%_ D_2cm_ | R_50%_ D_2cm_ | R_50%_ |
|  | PTV3 | Deviations | 0 | 2 | 1 | 1 | 0 | 1 | 0 | 0 | 0 | 0 |
|  |  | Parameters | - | PTV coverage R50 | R_50%_ (major) | R_50%_ | - | R_50%_ | - | - | - | - |
| Case 3 | PTV1 | Deviations | 0 | 2 | 0 | 1 | 0 | 0 | 0 | 1 | 0 | 0 |
|  |  | Parameters | - | PTV coverage R_50%_ | - | R_50%_ | - | - | - | D_2cm_ (major) | - | - |
| Case 4 | PTV1 | Deviations | 1 | 3 | 0 | 0 | 0 | 0 | 1 | 0 | 0 | 0 |
|  |  | Parameters | PTV coverage | PTV coverage R_50%_ D_2cm_ | - | - | - | - | D_2cm_ | - | - | - |
|  | PTV2 | Deviations | 0 | 2 | 0 | 0 | 0 | 0 | 0 | 0 | 0 | 0 |
|  |  | Parameters | - | PTV coverage R_50%_ | - | - | - | - | - | - | - | - |
|  | PTV3 | Deviations | 2 | 2 | 0 | 2 | 1 | 2 | 2 | 2 | 0 | 1 |
|  |  | Parameters | PTV coverage R_50%_ | PTV coverage R_50%_ | - | PTV coverage R_50%_ | PTV coverage (major) | PTV coverage R_50%_ | R_50%_ D_2cm_ (major) | PTV coverage, R_50%_ | - | R_50%_ |

Abbreviations: PTV, planning target volume.
^*^ Centers adopting PTV-EVAL in planning of all PTVs in case 4.
^†^ Centers adopting PTV-EVAL in planning of PTV3 in case 4.
^‡^ PTV contains multiple target volumes, thus R_50%_ and D_2cm_ deviations are exempted.

**Supplementary Figure 1. Study Scheme of the Study**

**
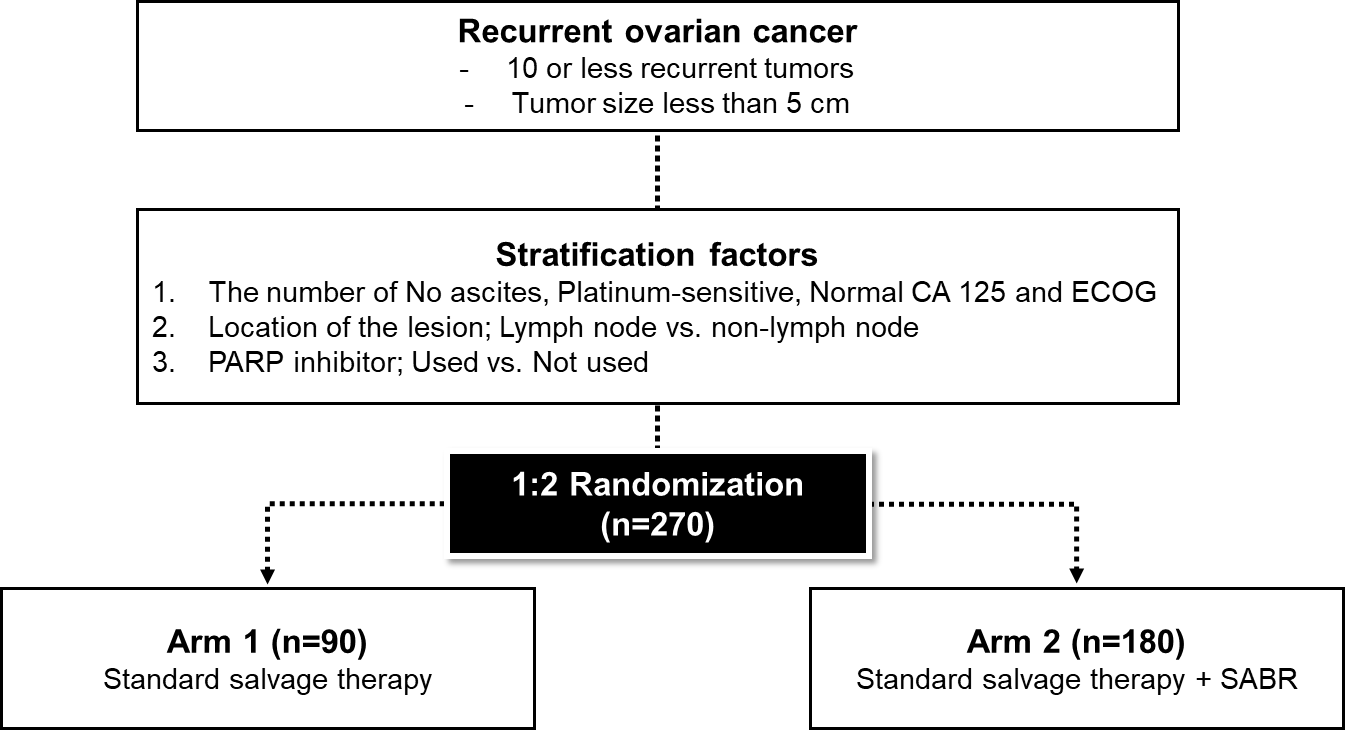
**

**Supplementary Figure 2.** **PET-CT scan at the time of salvage radiotherapy referral for Case 1, showing residual retroperitoneal LN metastases following chemotherapy.**


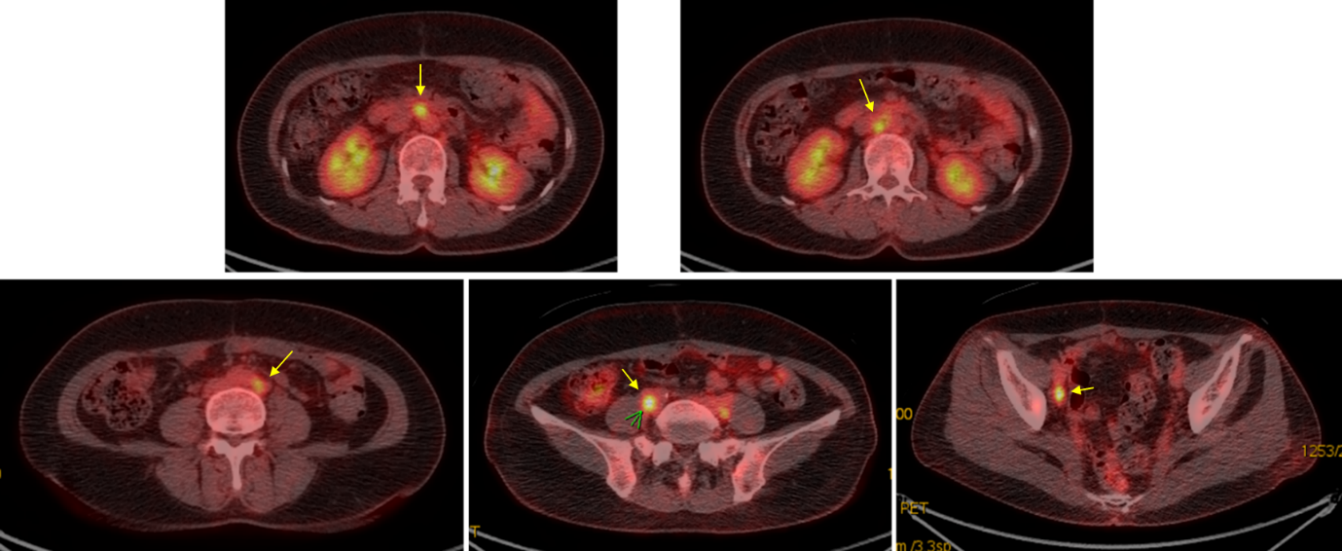


**Supplementary Figure 2.** **Chest CT scan of Case 2. Showing five metastatic lesions across both lungs at the time of the radiotherapy referral**


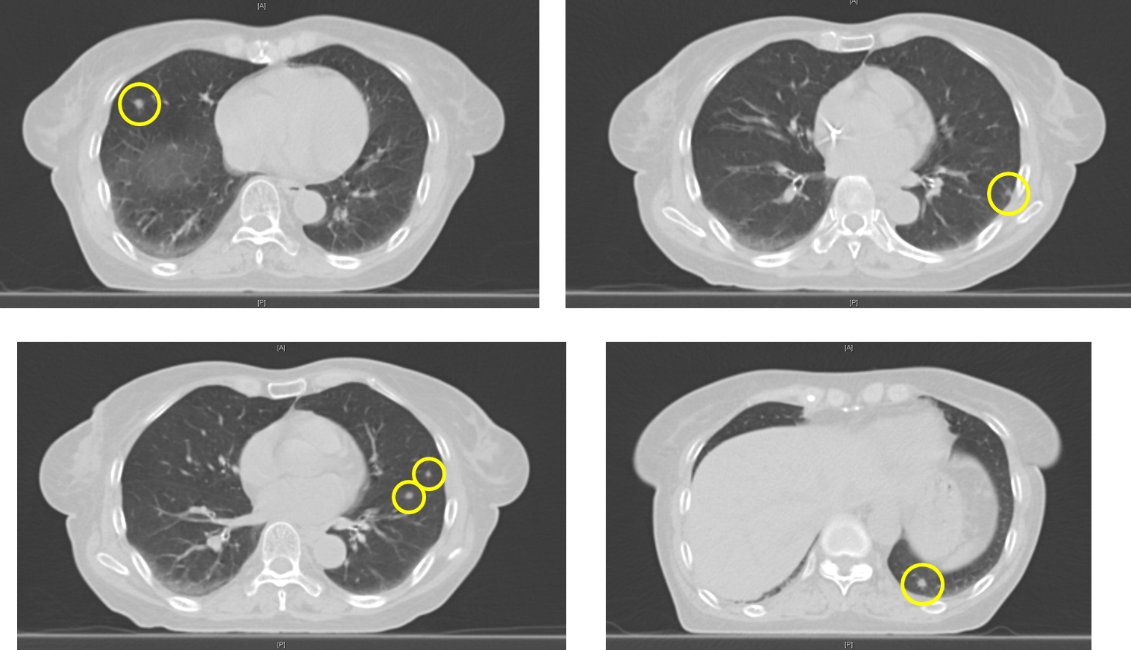


**Supplementary Figure 4. Abdominopelvic CT scan of Case 3, showing seeding metastasis regrowing between the liver and diaphragm.**


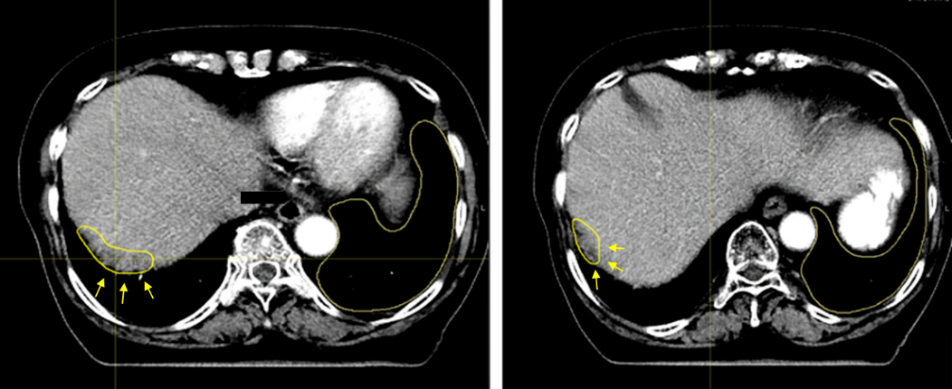


**Supplementary Figure 5. PET-CT scan of Case 4, showing multiple intraperitoneal seeding at the time of the salvage radiotherapy referral.**


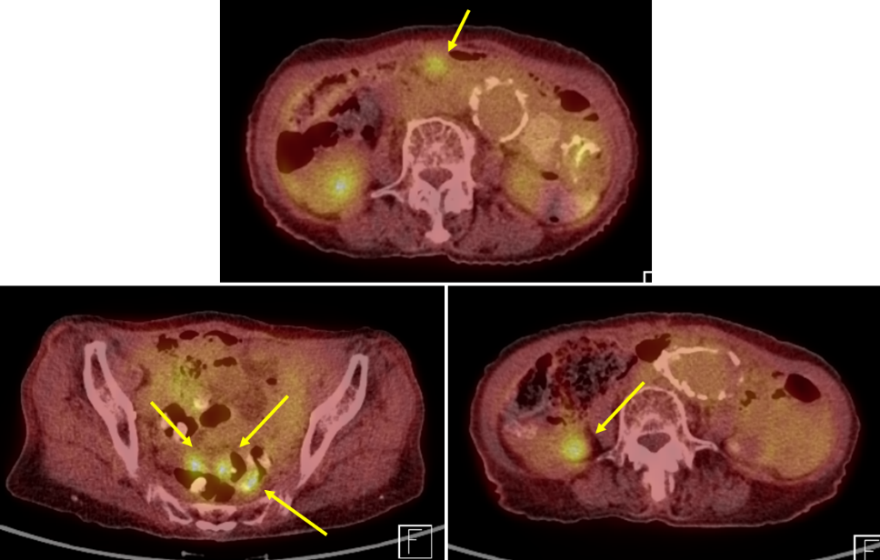


**Supplementary Figure 6. Comparison between PTV and PTV-EVAL. (A) The Planning Target Volume (PTV) is highlighted in blue. (B) PTV-EVAL, after subtracting organ-at-risk.**


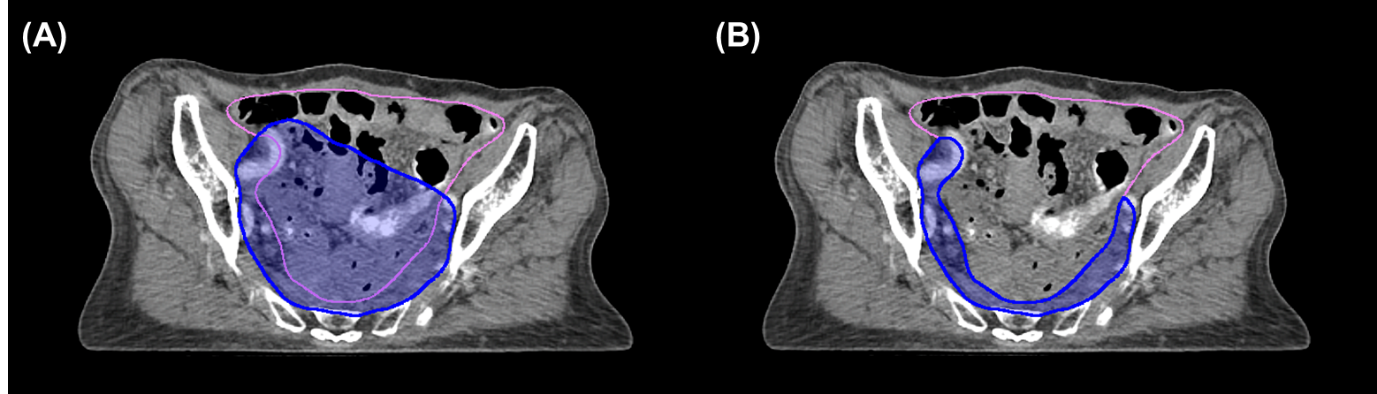

Supplement: Supplementary file 2 — Supplementary Material 2 [file 12885_2025_13892_MOESM2_ESM.docx]
